# A Microservices-Based Solution with Hybrid Communication for Energy Management in Smart Grid Environments

**DOI:** 10.3390/s26051714

**Published:** 2026-03-09

**Authors:** Artur F. S. Veloso, José V. Reis, Ricardo A. L. Rabelo

**Affiliations:** Department of Computing, Federal University of Piauí (UFPI), Teresina 64049-550, PI, Brazil; arturfdasveloso@ufpi.edu.br (A.F.S.V.); ricardoalr@ufpi.edu.br (R.A.L.R.)

**Keywords:** smart grid, energy management, demand response, load shifting, hybrid communication, LoRaWAN, LoRaMESH, Internet of Things (IoT), microservices architecture, predictive artificial intelligence, resilience, sustainability

## Abstract

The increasing variability of residential demand, combined with the expansion of distributed generation and electric vehicles, has introduced new challenges to the stability of Smart Grids (SGs). Centralized management models lack the flexibility required to operate under these conditions, reinforcing the need for scalable and data-driven architectures. This study proposes an energy management solution based on microservices, supported by hybrid communication in Low Power Wide Area Networks (LPWAN), integrating Long Range Wide Area Network (LoRaWAN) and LoRaMESH to enhance connectivity, local resilience, and reliability in data acquisition for Internet of Things (IoT) and Demand Response (DR) applications. A prototype composed of a Smart Meter (SM), a Data Aggregation Point (DAP), and a Concentrator (CON) was evaluated in a controlled environment, achieving Packet Delivery Rates above 97%, an average RSSI of −92 dBm, and a Signal-to-Noise Ratio close to 9 dB, validating the robustness of the hybrid communication. At a larger scale, data from 5567 households in the Low Carbon London (LCL) project were used to generate representative Load Profiles (LPs) through seven aggregation and clustering techniques, consistently identifying the 18:00–21:00 interval as the critical peak, with demand reaching up to 42% above the daily average. Fourteen load shifting algorithms were evaluated, and the Hybrid Adaptive Algorithm based on Intention and Resilience (HAAIR), proposed in this work, achieved the best overall performance with a 1.83% peak reduction, US$65.40 in cost savings, a reduction of 60 kg of CO_2_, a Comfort Loss Index of 0.04, resilience of 9.5, and reliability of 0.98. The results demonstrate that the integration of hybrid LPWAN communication, modular microservice-based architecture, and adaptive DR strategies driven by Artificial Intelligence (AI) represents a promising pathway toward scalable, resilient, and energy-efficient SGs.

## 1. Introduction

The global energy sector is undergoing a significant transformation driven by the principles of decarbonization, decentralization, and digitalization [1,2,3,4,5]. This process is motivated both by the need to address challenges such as climate change and instabilities in energy supply [6,7,8] and by the continuous increase in electricity consumption resulting from sectoral electrification.

The growing integration of intermittent renewable sources, particularly solar and wind [9,10,11], intensifies supply variability, while short periods of high demand place pressure on infrastructure, requiring the activation of flexible and higher-cost generation units [12].

In this context, Demand Response (DR) has become an important mechanism for mitigating stress on the grid by adjusting consumer demand according to operational conditions [13,14,15,16]. Among DR strategies, Load Shifting stands out for redistributing consumption from peak periods to times of lower demand [17,18]. However, the practical implementation of this mechanism depends on bidirectional metering and communication infrastructure that is low-cost and reliable enough to support actions at the residential scale.

The digitalization of power networks through Smart Grid (SG) and Internet of Things (IoT) [3,19,20], together with the growing presence of Recursos Energéticos Distribuídos (DER) [21,22], offers new opportunities to operationalize DR at scale. However, the existing literature remains fragmented: studies focus separately on IoT-based metering [23,24], microservices platforms [25,26], or optimization algorithms [14] but rarely propose an integrated and operationally validated solution that combines reliable communication, analytical scalability, and intelligent decision-making. This gap hinders the practical adoption of DR in real-world environments [21,22].

**Figure 1:**
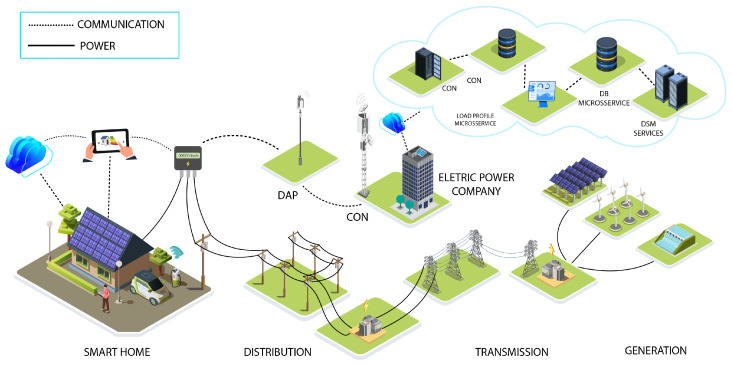
Smart Grid Architecture.

The high-level architecture considered in this work, illustrated in Figure 1, integrates three layers: residential devices, hybrid communication, and analytical services. In the home environment, Smart Meter (SM), photovoltaic systems, electric vehicle chargers, and user interfaces connect to a Data Aggregation Points (DAP), which is responsible for local communication. The DAP communicates with a Concentrators (CON) using both Long Range Wide Area Network (LoRaWAN) and Long Range Mesh Network (LoRaMESH) simultaneously, increasing resilience and mitigating coverage limitations typically found in single-topology Low Power Wide Area Network (LPWAN) architectures. The CONs forward data to the utility company and to a cloud platform, where microservices execute Load Profile (LP) generation, data storage, and DR strategies. This architecture supports two core functions: (i) automatic identification of critical peak periods and (ii) selection of eligible consumers for Load Shifting.

To validate the feasibility of the proposal, a physical prototype was developed, composed of a SM, a residential DAP, and DAP/CON communication implemented with ESP32 integrating LoRaWAN and LoRaMESH. The CON was implemented using a Raspberry Pi 3 is designed by the Raspberry Pi Foundation and manufactured primarily at the Sony UK Technology Centre in Pencoed, Wales, UK, with a LoRaWAN gateway, and the infrastructure used The Things Network (TTN). Backend microservices were implemented in Python 3.11.6 and Django 3.2.25 and deployed on an Amazon Web Services (AWS) server. This environment demonstrated consistent operation of the architecture in a controlled scenario.

In parallel, a large-scale analytical validation was conducted using the Low Carbon London (LCL) dataset, containing real consumption profiles from 5567 households. This dataset enabled the generation of LPs, peak detection, and DR simulations, complementing the experimental validation with quantitative analyses.

The main contributions of this work are as follows:

- Proposal of an end-to-end architecture that integrates hybrid LoRaWAN/LoRaMESH communication, microservices, and energy analytics within a unified operational ecosystem.
- Practical implementation and validation of hybrid communication to enhance resilience and reliability in data acquisition.
- Development of a modular microservices platform for LP generation and peak detection.
- Proposal and validation of the Hybrid Adaptive Algorithm based on Intention and Resilience (HAAIR) algorithm, which incorporates behavioral intention and network resilience into the DR decision-making process.
- Experimental evaluation combining (i) a physical prototype and (ii) large-scale simulation with real LCL data.

These contributions guide the following research questions:

- RQ1: Does a microservices-based architecture supported by hybrid LoRaWAN/LoRaMESH communication provide the scalability and resilience required for real-time DR operations?
- RQ2: Does the HAAIR algorithm, by incorporating behavioral intention and network resilience, improve multiobjective DR performance (peak reduction, cost savings, and Command-Line Interface (CLI)) compared to state-of-the-art methods?

To address these questions, the study is organized into three steps: (i) evaluation of hybrid communication (RQ1); (ii) analysis of LP generation and peak detection (support for RQ2); and (iii) comparison of DR strategies, including the HAAIR algorithm (validation of RQ2). The remainder of the article is structured as follows: Section 2 reviews the state of the art; Section 3 presents the proposed architecture; Section 4 describes the experimental setup and results; Section 5 discusses the implications; and Section 6 concludes the study.

## 2. Related Work

The evolution of SG involves multiple technological layers that interact in a complementary manner, including communication, metering, large-scale analytics, and intelligent decision-making. Although the literature presents significant advances in each of these domains, a notable gap remains in solutions that integrate, in a cohesive and operationally validated manner, hybrid communication, intelligent devices, and advanced DR algorithms. This section critically reviews the state of the art across three main axes: (i) communication technologies, (ii) SM architectures, and (iii) microservices and Artificial Intelligence (AI) applied to SG. The analysis highlights recurring limitations and motivates the integrated proposal developed in this work.

### 2.1. Communication Technologies for SG

Communication is the foundational element of any SG infrastructure, as it supports continuous monitoring, distributed control, and the reliable execution of DR mechanisms. However, no single technology simultaneously meets the requirements for scalability, low cost, reduced latency, reliability, and fault tolerance. The literature presents a broad range of technologies, summarized in Table 1, whose critical analysis is expanded below. Wired solutions such as Power Line Communication (PLC), Ethernet, and optical fiber remain fundamental backbones. PLC [27] leverages existing electrical infrastructure and offers reduced costs, but suffers from impulsive noise and variable impedance, which compromise time-sensitive applications. Ethernet [28] ensures high bandwidth, low latency, and stability, though it is restricted to controlled and short-range environments. Optical fiber [29] provides immunity to interference and extremely high data rates, making it suitable for utility backbones but economically impractical in residential or rural scenarios.

Short- and medium-range wireless technologies include WiFi [30], Bluetooth Low Energy (BLE) [31], Zigbee [32], and Z-Wave [33]. WiFi offers high throughput, but with limited range and high sensitivity to obstacles. Zigbee and Z-Wave enable mesh networking with low energy consumption, making them suitable for home automation and IoT systems, though insufficient for high-density networks or long distances. WirelessHART [34] provides high robustness in industrial environments but requires complex deployments and specialized hardware. Cellular networks (2G–5G) [35] offer wide coverage and low latency, particularly with 5G, enabling near real-time control. However, they depend on telecom operators, incur recurring costs, and may exceed the requirements for sporadic sensing applications. LPWAN technologies built on telecom infrastructure, such as Narrowband Internet of Things (NB-IoT) and Long-Term Evolution Machine (LTE-M) [36,37], expand coverage and energy efficiency, though they still require contracted services and may experience degradation in underground or highly built environments.

Independent LPWAN networks have gained significant attention, especially LoRaWAN [36], which combines long range, low power consumption, and unlicensed spectrum. However, its star topology introduces a single point of failure: gateway unavailability compromises all local communication. LoRaMESH [38] mitigates this limitation through adaptive routing and multi-hop self-reconfiguration, though challenges persist regarding coordination, energy management, and scalability in dense topologies. Other approaches, such as Sigfox [39], maximize energy efficiency but impose extreme restrictions on payload size and throughput.

The literature shows that no single technology solves the trade-off between range, latency, resilience, and operational cost. For this reason, hybrid architectures have gained increasing attention. Prior works have explored combinations such as NB-IoT + GPRS [40], WiFi + PLC [41], Zigbee + Long Range (LoRa) [42], and LTE-M + BLE [43]. However, few have been validated in real SG environments, with most remaining confined to simulations or controlled testbeds. The hybrid integration of LoRaWAN + LoRaMESH investigated in [44,45,46], and expanded in this work, represents one of the most promising approaches, as it combines long-range communication with local resilience and autonomy in both urban and rural environments. In this article, this approach is experimentally validated using proprietary SM, DAP, and CON devices, demonstrating real redundancy, fault tolerance, and support for upper-layer DR analytics and control.

**Table 1:** Communication Technologies Applicable to SG.

Ref.	Category	Technology	Frequency (MHz)	Data Rate	Network Specifications	Typical Distance	Average Cost	Key Features
[27]	Wired	PLC	1–30 (NB)/100–500 (BB)	10 kbps–1 Mbps	Power line/point-to-point or multipoint	1–3 km (low-voltage grid)	Low	Utilizes power infrastructure, susceptible to noise
[28]	Wired	Ethernet	10/100/1000 MHz	Up to 1 Gbps	Switched/point-to-point	100 m (without repeater)	Medium	High stability and bandwidth
[29]	Wired	Optical Fiber	Light frequency	Gbps–Tbps	Point-to-point/ring	>10 km	High	Immune to interference, high installation cost
[47]	Wired	RS-485/Modbus	-	Up to 10 Mbps	Shared bus	1.2 km	Low	Simple and robust, widely used in automation
[30]	Wireless	Wi-Fi (802.11 b/g/n)	2.4/5 GHz	11 Mbps–600 Mbps	Point-to-point/Access Point	30–100 m	Low	High data rate, limited by obstacles
[31]	Wireless	Bluetooth (BLE)	2.4 GHz	Up to 2 Mbps	Point-to-point/limited mesh	10–30 m	Low	Low power, local communication
[32]	Wireless	Zigbee (802.15.4)	2.4 GHz	20–250 kbps	Mesh, star, tree	10–100 m	Low	Low power, suited for residential/industrial IoT
[33]	Wireless	Z-Wave	868/915 MHz	Up to 100 kbps	Mesh	30–100 m	Medium	High reliability, home automation focus
[34]	Wireless	WirelessHART	2.4 GHz	250 kbps	Industrial mesh	100 m (per hop)	Medium	Robust in industrial environments
[35]	Wireless	Cellular (2G/3G/4G/5G)	800/900/1800/2600 MHz	50 kbps–>1 Gbps	Star via cell tower	1–20 km (macrocell)	Medium–High	Wide coverage, cost depends on usage
[36]	LPWAN	LoRaWAN	868/915 MHz	0.3–50 kbps	Star	2–15 km (urban/rural)	Low	Long range, low power consumption
[38]	LPWAN	LoRaMESH	868/915 MHz	0.3–50 kbps	Adaptive mesh	1–5 km per hop	Low	High local resilience, self-organizing
[39]	LPWAN	Sigfox	868/915 MHz	Up to 100 bps uplink	Star (via operator network)	3–10 km (urban)/50 rural	Low	Ultra-low power, very limited payload
[36]	LPWAN	NB-IoT	<1 GHz	Up to 250 kbps	Star (via carrier)	1–10 km	Medium	Deep coverage and carrier support
[37]	LPWAN	LTE-M (Cat-M1)	<1 GHz	Up to 1 Mbps	Star (via carrier)	1–10 km	Medium–High	Voice/mobility support, costlier than NB-IoT
[48]	LPWAN	GPRS (2.5G)	900/1800 MHz	56–114 kbps	Star (via carrier)	5–10 km	Medium	Legacy systems still widely supported
[44,45,46] and This Work	Hybrid	LoRaWAN + LoRaMESH	868/915 MHz	0.3–50 kbps	Star + Mesh	2–15 km + local redundancy	Low–Medium	Combines long range with local resilience
[40]	Hybrid	NB-IoT + GPRS	<1 GHz/900 MHz	50–250 kbps	Star with fallback	1–10 km	Medium	Legacy redundancy and wide coverage
[41]	Hybrid	Wi-Fi + PLC	2.4 GHz + 2–30 MHz	Up to 100 Mbps	Wi-Fi with power-line fallback	30–100 m	Medium	Continues operating without wireless network
[42]	Hybrid	Zigbee + LoRa	2.4 GHz + 868 MHz	250 kbps + 0.3 kbps	Local mesh + long-range backbone	10–100 m + 2–10 km	Medium	Industrial-grade hybrid backbone
[43]	Hybrid	LTE-M + BLE	<1 GHz + 2.4 GHz	1 Mbps + 2 Mbps	Star + local	1–10 km + 30 m	Medium–High	Mobile communication + secure local provisioning

Numerous studies have advanced the use of IoT for monitoring, control, and automation in SG, exploring solutions from low-cost sensors to modular architectures with multiple communication interfaces, as described in Table 1. However, a detailed analysis reveals important limitations related to scalability, communication resilience, and operational integration with large-scale analytical services. Proposals based on simple microcontrollers, such as Arduino UNO and ESP8266, demonstrate feasibility for initial experimentation but lack the processing capacity, security, and robustness required for complex residential and industrial scenarios. Other solutions rely on isolated communication protocols such as Wireless Fidelity (WiFi), BLE, PLC, or LPWAN technologies, particularly LoRaWAN, which offer an attractive balance between range and power consumption but frequently encounter structural limitations in topology, latency, and fault tolerance. Even systems based on NB-IoT and LTE-M, while providing broad coverage, introduce dependence on external operators, increasing operational costs and reducing control over infrastructure, a critical issue for DR applications.

When examining data acquisition architectures in the literature, it becomes evident that most studies focus on SM device functionality but devote little attention to network robustness in scenarios involving local failures, congestion, interference, or temporary gateway unavailability. Few research works propose redundancy or self-organization mechanisms in the communication layer, and the majority depend on rigid star topologies, which are particularly vulnerable in dense urban SG environments or rural regions with limited infrastructure. Although some studies mention mesh networks or hybrid solutions, they seldom provide experimental validation with physical devices in real environments or discuss how different technologies interact under adverse conditions such as link loss, shadowing, or multipath effects. This gap compromises the reliability of collected data and limits the applicability of devices in advanced optimization and control algorithms.

Another recurring issue is the frequent disconnect between the IoT layer and analytics platforms based on microservices. Many studies evaluate device performance in isolation or in small-scale testbeds but do not integrate these measurements into real data-processing pipelines, LP generation, or DR strategies. This absence of integration makes it difficult to reproduce practical scenarios in which thousands of readings must flow continuously and uninterrupted to decision-making modules. In the literature, most solutions remain dependent on monolithic architectures with limited scalability, poor updateability, and weak interoperability among heterogeneous devices. Furthermore, few studies explicitly discuss how network decisions, such as routing, latency, packet loss, and Received Signal Strength Indicator (RSSI) variability, affect the effectiveness of cloud-based analytical services. Thus, despite individual advances, the field lacks an approach that unifies resilient hybrid communication, interoperable intelligent devices, and seamless integration with energy-related microservices.

These observations reveal that although there is considerable diversity in IoT solutions for SG, a significant gap remains in the development and validation of architectures capable of operating robustly, scalably, and in a coordinated manner in real environments. The literature particularly lacks models that combine hybrid topologies, local redundancy, multi-hop operation, and direct integration with large-scale analytical systems. These limitations motivate the need for an IoT architecture that provides stable connectivity even under failures, supports large volumes of heterogeneous devices, and guarantees the data quality required by advanced DR algorithms. This is precisely the gap addressed by the present work, which aims to overcome these limitations identified in the state of the art.

### 2.2. Smart Meter Implementation

The literature presents a broad and heterogeneous set of SM architectures, reflecting different design priorities ranging from low cost to high robustness for industrial applications. However, a systematic analysis of these proposals reveals consistent limitations related to scalability, reliability, and operational integration with SG platforms. Table 2 summarizes the main studies and highlights this diversity of approaches.

Low-cost prototypes based on microcontrollers such as Arduino UNO and Espressif ESP8266 Microcontroller (ESP8266), often combined with simple sensors (Current Transformer (CT), resistive dividers), are widely used in experimental studies. These solutions demonstrate initial feasibility for residential energy monitoring but remain constrained by structural limitations: low processing capacity, absence of robust security mechanisms, lack of interoperability, and limited performance under real-world conditions of electromagnetic noise, shadowing, or abrupt load variations. Such limitations hinder their adoption in large-scale SG environments, where high reliability and communication stability are essential requirements.

More advanced architectures employ NB-IoT, LTE-M, or traditional cellular modules to achieve greater range and stability. While these systems exhibit adequate performance in urban scenarios, they rely heavily on telecom infrastructure operated by third parties, which leads to recurring costs, variable latency, and reduced control over service availability. Furthermore, the integration of these technologies with private SG networks is often limited, impairing the execution of critical functionalities such as near real-time DR.

Other approaches use modular platforms such as Raspberry Pi Zero W combined with Zigbee or WiFi networks. These solutions provide increased computational capacity and support integration with residential gateways, but still lack mesh-level resilience mechanisms and remain vulnerable to single-point connectivity failures. Even industrial-grade LoRaWAN solutions, as reported in [49], although robust and suitable for factory environments, adopt strictly star topologies and rarely incorporate local redundancy or self-reconfiguration capabilities, which are critical for continuous operation in distributed and dense SG environments.

A comparison of recent studies shows that few SM architectures propose effective mechanisms to address two central challenges: (i) independence from external infrastructure, and (ii) maintenance of connectivity under local failures, congestion, or physical variability of the environment. The literature also lacks experimental validations in hybrid environments with multiple communication technologies operating simultaneously, a fundamental requirement for complex urban settings or networks with high device density. Finally, most works evaluate their prototypes in isolation, without robust integration with microservices, Meter Data Management System (MDMS), Non-relational Database (NoSQL) databases, or distributed analytics platforms, limiting the extrapolation of results to operational SG contexts.

These limitations reveal an important gap in the state of the art: the absence of SM architectures that combine low energy consumption, high scalability, hybrid communication with local redundancy, and self-reconfiguration capabilities. Existing studies advance partially toward these characteristics but generally do not offer solutions capable of operating resiliently and continuously in large-scale networks, especially in heterogeneous and failure-prone environments.

**Table 2:** Comparative Architectures of SM for SG.

Ref.	Microcontroller/SoC	Sensors Used	Communication Interface(s)	Measurement Parameters	Topology	Power Supply	Deployment Scope	Key Contributions
[46]	ESP32	Voltage/Current (ACS712)	Wi-Fi, LoRaWAN, LoRaMESH, BLE, GSM	Energy, Power, RMS, PF	Modular + Hybrid	AC/DC Converter	Real residential + DAP	Hybrid LoRaWAN + MESH, real DAP deployment
[50]	Arduino UNO	Voltage divider + CT sensor	Wi-Fi	Voltage, Current, Energy	Star (Wi-Fi)	Grid-powered	Low-cost home pilot	Affordable Wi-Fi-based SM prototype
[36]	STM32F103	Hall-effect sensors	NB-IoT	Energy, Voltage, Frequency	Star (NB-IoT)	Battery + solar	Field testing (urban)	NB-IoT energy monitoring system
[51]	ESP8266	Current transformer + ZMPT101B	Wi-Fi + MQTT	Voltage, Current, kWh	Centralized cloud	USB/AC adapter	University labs	Cloud-integrated metering IoT prototype
[42]	Raspberry Pi Zero W	CT sensor + Voltage sensor	Zigbee + Wi-Fi	Voltage, Current, Peak Demand	Zigbee mesh + Gateway	AC via SMPS	Testbed environment	Zigbee–Wi-Fi bridging for SG data
[49]	ESP32 + co-processors	Industrial-grade sensors	LoRaWAN	Voltage, Current, Power Factor, THD	Star	AC/solar + UPS	Factory deployment	Modular multi-sensor LoRaWAN-based SM

In addition, frequent disconnection is observed between edge devices and upper processing layers. Few studies natively integrate their SM with data pipelines based on microservices, hindering real-time execution of functionalities such as LP generation, automatic peak detection, or orchestration of DR algorithms. In many cases, SM operate only as passive data sources, without support for adaptive operations or reliable bidirectional communication, both essential requirements for modern distributed control and optimization strategies.

Finally, despite the growing adoption of LPWAN technologies, the literature presents few studies that validate truly hybrid architectures capable of combining the long-range capabilities of LoRaWAN with local resilience mechanisms such as multi-hop routing and self-recovery present in LoRaMESH networks. In general, evaluations remain limited to simulations or small-scale scenarios, failing to investigate how such solutions behave under real requirements of latency, packet loss, or high device density.

These observations reveal a clear opportunity for advancements: the development and validation of SM architectures that integrate complementary technologies, operate autonomously even under adverse conditions, and connect directly to scalable analytical ecosystems. This study builds upon these gaps identified in the state of the art to support the development of an integrated approach combining hybrid communication, intelligent metering, and analytical support tailored to SG operations.

### 2.3. Microservices and Artificial Intelligence in SG

The evolution of software architectures for SG has been strongly influenced by the parallel advancement of microservice-based infrastructures and AI algorithms. These two technological axes have become central in the construction of more flexible, scalable, and responsive power systems. However, the literature shows that such advances often occur in a fragmented manner: studies exploring load forecasting, fault detection, cybersecurity, or DR optimization tend to propose isolated solutions that rely on monolithic or experimental platforms, without integration with the broader digital ecosystem of the power grid, as detailed in Table 3.

Recent studies in load forecasting have explored architectures based on Deep Neural Networks (DNN), Long Short-Term Memory Network (LSTM), Gated Recurrent Unit (GRU), AutoRegressive Integrated Moving Average (ARIMA), Extreme Gradient Boosting (XGBoost), and hybrid models [52]. These approaches significantly improve accuracy for both short- and long-term horizons, but they face challenges related to the need for large quantities of high-quality data, sensitivity to anomalies, and high computational cost in large-scale deployments. In DR, studies such as [53] apply reinforcement learning algorithms including Proximal Policy Optimization (PPO), Deep Deterministic Policy Gradient (DDPG), and Q-Learning Reinforcement Algorithm (Q-Learning), often in multi-agent scenarios. Although these methods achieve meaningful peak reductions, they still struggle with convergence issues, agent coordination, and reliance on simulated datasets, limiting their operational applicability.

Fault detection and anomaly identification in SG have been explored using Convolutional Neural Network (CNN), Support Vector Machine (SVM), Random Forest (RF), and autoencoders, as demonstrated in [54,55]. While these solutions advance predictive maintenance and non-technical loss detection, they face limitations stemming from scarcity of labeled data, class imbalance, and high false-positive rates. In challenges related to voltage and frequency stability, models based on Model Predictive Control (MPC), fuzzy logic, and reinforced AI [56] show promising results, but they require low-latency communication infrastructure and considerable computational capacity. In renewable generation forecasting, authors such as [57] employ hybrid models (LSTM, CNN-LSTM, Random Forest) that depend on highly precise meteorological data.

**Table 3:** Comparison of Microservices and AI Algorithms Applied to SG.

Ref.	Microservice	Primary Function in SG	Typical AI Algorithms Used	Benefits	Challenges/Limitations
[52]	Load Forecasting Service	Predict short-term and long-term energy consumption patterns.	ANN, LSTM, GRU, ARIMA, XGBoost, Transformer-based models.	Improves Demand-side Management, optimizes energy distribution, supports dynamic pricing.	Requires high-quality historical data; sensitive to anomalies; high computational cost for deep models.
[53]	Demand Response Optimization Service	Manage and optimize load shifting based on consumer flexibility and price signals.	Reinforcement Learning (Q-Learning, PPO, DDPG), Multi-Agent RL, Evolutionary Algorithms (NSGA-II).	Reduces peak demand, improves grid stability, minimizes operational costs.	Complexity in multi-agent coordination; requires real-time adaptability and fast convergence.
[54]	Fault Detection and Diagnostics Service	Identify and classify faults in grid equipment and communication nodes.	CNN, SVM, Random Forest, Autoencoders for anomaly detection.	Enables predictive maintenance, reduces downtime, increases reliability.	Needs labeled fault datasets; false positives can lead to unnecessary interventions.
[55]	Energy Theft Detection Service	Detect non-technical losses and fraudulent energy usage.	Decision Trees, SVM, Deep Learning, Graph Neural Networks (GNN).	Reduces financial losses, improves grid security.	Imbalanced datasets; risk of misclassification; privacy concerns.
[56]	Voltage and Frequency Stability Service	Monitor and control voltage/frequency deviations in real-time.	Model Predictive Control (MPC), Reinforcement Learning, Fuzzy Logic.	Enhances power quality, prevents blackouts, supports renewable integration.	Requires fast response and robust communication; computationally intensive.
[57]	Renewable Energy Forecasting Service	Predict generation from solar, wind, and other renewables.	LSTM, CNN-LSTM, Random Forest, Gradient Boosting, Hybrid Deep Models.	Facilitates integration of intermittent sources, improves dispatch planning.	Dependent on accurate weather data; uncertainty in forecasts.
[58]	Electric Vehicle (EV) Charging Optimization Service	Schedule and optimize EV charging to minimize grid impact.	Multi-Objective Optimization, RL, Genetic Algorithms, Swarm Intelligence.	Balances load, reduces congestion, supports V2G strategies.	Dynamic user behavior; communication latency; scalability issues.
[59]	Market Pricing and Trading Service	Predict and optimize energy prices for market operations.	Time-Series Forecasting (LSTM, Prophet), Bayesian Networks, RL-based trading agents.	Improves profitability for operators, supports real-time pricing strategies.	Market volatility; requires integration with external economic indicators.
[60]	Grid Topology Reconfiguration Service	Dynamically reconfigure the grid to improve reliability and minimize losses.	Graph Neural Networks, Evolutionary Algorithms, MILP with AI enhancements.	Enhances resilience, reduces transmission losses, adapts to changing conditions.	Complexity in large-scale networks; real-time constraints.
[61]	Cybersecurity Threat Detection Service	Monitor and mitigate cyber threats in SG communication.	Deep Autoencoders, Intrusion Detection with ML (Isolation Forest, LSTM), Federated Learning.	Increases network security, mitigates cyberat-tacks, protects critical infrastructure.	Evolving attack patterns; need for distributed security; high false positive rates.

Additional applications include optimization of electric vehicle (Electric Vehicle (EV)) charging using multi-objective algorithms, Reinforcement Learning (RL), swarm optimization, and Vehicle-to-Grid (V2G) strategies [58], along with advanced pricing and energy trading mechanisms employing temporal models such as Facebook Prophet Forecasting Model (Prophet), LSTM, and RL agents [59]. Studies have also explored dynamic reconfiguration of grid topology through Graph Neural Network (GNN), evolutionary algorithms, and Mixed Integer Linear Programming (MILP) formulations [60], as well as cyberthreat detection mechanisms with deep autoencoders, federated learning, and intrusion detection systems (Intrusion Detection System (IDS)) [61]. Despite these notable advances, few works propose cohesive software platforms capable of unifying these services as scalable, interoperable microservices within the context of SG.

A comprehensive analysis of the literature reveals three main gaps. First, most studies focus on AI-based solutions executed in isolation, often validated solely through simulations, without direct integration with physical devices, communication protocols, or real operational requirements of power networks. Second, microservice platforms are mentioned but rarely implemented to handle continuous energy data flows, IoT compatibility, scalability requirements, and orchestration of near real-time decision-making. Third, optimization algorithms used in DR generally overlook behavioral factors and dynamic communication constraints, relying on fixed cost functions or simplified decision models.

In this context, the present work builds on lessons extracted from the state of the art and proposes to address these gaps simultaneously. Unlike the fragmented literature, this study adopts a holistic approach by integrating (i) a modular set of microservices designed for ingestion, analysis, prediction, and control; (ii) fault-tolerant hybrid communication between SM, DAP, and CON; and (iii) intelligent decision-making mechanisms that incorporate user behavioral intention and network resilience. This approach provides a missing link between advanced AI models and the physical, behavioral, and communication constraints that characterize operational SG environments. By aligning forecasting, adaptive control, and distributed execution, this work advances the literature by proposing an integrated system capable of supporting near real-time DR decisions with higher reliability and lower user impact.

In addition to the technological aspects shaping the evolution of SG, there is growing interest in understanding how real consumers respond to price signals and how such behaviors influence the effectiveness of DR programs. Dynamic pricing schemes such as Dynamic Time-of-Use (dToU) and Real-Time Pricing (RTP) have been extensively investigated as mechanisms to incentivize load shifting, yet studies consistently show that consumer decision-making under these regimes remains complex, nonlinear, and highly heterogeneous [62]. Tariff responsiveness is influenced by factors such as prior experience with energy management, clarity of price signals, the level of automation available in the household, and the presence of feedback mechanisms, including real-time consumption visualization. Critically, research reports asymmetric behavioral responses: consumers tend to react strongly only to extreme peak prices, showing minimal sensitivity to moderate variations; furthermore, most users shift loads only when automated systems intervene [63]. These findings reinforce that dynamic tariffs alone are insufficient to sustain robust DR programs. The literature converges on the need for intelligent mechanisms capable of learning individual preferences, routines, and constraints, coordinating appliance rescheduling in a personalized manner while preserving user comfort. This trend directly supports the design philosophy of the HAAIR algorithm, which combines adaptive resilience with behavioral consistency in tariff-oriented interventions.

A second emerging research axis relates to the Energy Routing Demand-Constrained Multi-Stage Task (ERDCMST), part of a family of multi-stage routing and energy allocation problems with constraints, in which a limited resource must be distributed over time while respecting priorities, operating windows, and energy-shifting limits [64]. ERDCMST-derived models extend classical formulations by incorporating temporal coupling and multiple competing objectives, making them highly suitable for DR applications in which loads must be shifted across intervals while meeting peak-reduction targets, acceptable discomfort thresholds, and daily operating limits. Recent works employ evolutionary algorithms, robust optimization, and RL to solve ERDCMST variants, demonstrating promising performance in multi-objective and uncertainty-rich environments. The HAAIR algorithm proposed in this work conceptually aligns with this tradition by integrating, for the first time, adaptive estimation of user intention and network resilience metrics, expanding traditional formulations with behavior-sensitive mechanisms, an element still largely absent in most optimization-based approaches [65].

Finally, the literature highlights that although advances in AI and microservice-based platforms have expanded forecasting, control, and automation capabilities in SG, significant gaps remain in achieving real-time multi-objective coordination under adverse operational conditions. Most optimization algorithms employed in DR explicitly ignore behavioral factors, assume static comfort models, and rely on rigid objective targets that, in practice, lead to user disengagement. At the same time, control strategies often overlook the resilience of the communication layer, operating under the idealized assumption of stable, low-latency networks. These limitations compromise both the reliability of DR actions and user acceptance. The present work seeks to fill this gap by integrating hybrid communication resilience, based on LoRaWAN and LoRaMESH, with predicted user intention, represented mathematically as $I_{i,t}$, within a modular microservice platform. Such integration enables adaptive decision-making capable of balancing economic, operational, and behavioral objectives in real time. This approach not only addresses limitations identified in the literature but also introduces a capability absent from previous works: maximizing system-wide benefits while rigorously minimizing perceived user discomfort, one of the main barriers to large-scale DR adoption.

In summary, despite progress in communication, IoT, SM architectures, microservices, AI algorithms, and behavioral models, a substantial gap remains in the literature concerning systemic integration, operational validation, and advanced behavioral modeling applied to DR. Foundational studies on adoption, intention, asymmetric response, and temporal energy constraints are rarely incorporated into complete, field-tested solutions. Incorporating these references, especially those related to adoption behavior, decision-making under dynamic tariffs, and ERDCMST formulations, is essential for rigorous contextualization of the domain. This analysis reinforces that the scientific landscape still lacks an architecture that is truly integrated, resilient, cognitive, and field-validated, a gap that the present work aims to address.

## 3. Proposed Microservices-Based Solution with Hybrid Communication

This section presents an integrated architecture that combines hybrid communication, intelligent IoT devices, and a modular microservice platform to support SG operations. The proposed solution addresses limitations identified in the literature, which frequently treats system components in isolation and without operational validation. Wired technologies offer high reliability but have elevated deployment costs and limited flexibility in residential scenarios. Conversely, wireless and LPWAN solutions such as LoRaWAN provide long-range connectivity but exhibit structural limitations, including dependence on a star topology, susceptibility to physical obstructions, and variability in Packet Delivery Ratio (PDR) under dense environments. These factors hinder continuous data acquisition, which is essential for DR applications.

The proposed solution overcomes these limitations by integrating LoRaWAN and LoRaMESH into a hybrid communication model. LoRaWAN is used for long-range connectivity, while LoRaMESH provides redundancy and alternative routing paths at the DAP layer, increasing resilience and reducing reliance on a single link to the CON. This combination enhances communication stability in urban scenarios and enables reliable execution of analytical and control services in near real time. Unlike approaches limited to simulations, the architecture was validated in a real environment using SMs, DAPs, and CONs integrated into the microservice platform. The resulting system performs data collection, processing, profile generation, peak detection, and DR optimization, including support for the HAAIR model. The following subsections detail the main components of this solution.

### 3.1. Overview and System Architecture

The objective of this research is to design, implement, and validate a robust and scalable architectural framework capable of meeting the communication, processing, and operational requirements of SG environments. To this end, functional prototypes of the SM, DAP, and CON devices were developed, enabling the evaluation of the proposed hybrid communication model under real-world conditions. In parallel, a modular microservice platform was constructed and organized into stages that include time-series preprocessing, LP generation, comparison between monolithic and microservice-based architectures, and the application of clustering algorithms to characterize consumption profiles. The integration between physical devices and analytical components ensures data-flow consistency, interoperability, and continuous operational capability.

To validate the system at scale, the LCL dataset made available by UK Power Networks was employed (https://data.london.gov.uk/dataset/smartmeter-energy-use-data-in-london-households, accessed on 24 January 2025). This dataset contains consumption records from more than 5567 London households, amounting to approximately 10 GB distributed across 167 million measurements collected every 30 min between 2011 and 2014. It includes around 1100 consumers under dToU tariffs, used for DR simulations, and approximately 4567 consumers under fixed tariffs. The implementation of the SM, DAP, and CON devices relied on low-cost open-source platforms, ensuring flexibility and adherence to the proposed hybrid communication infrastructure. The following subsections detail the role and operation of each element within the microservice-oriented architecture.

### 3.2. Smart Meter Design and Implementation

**Figure 2:**
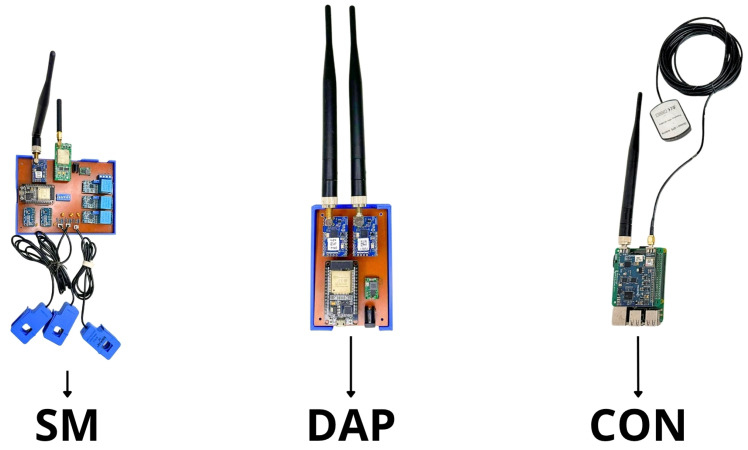
Physical prototypes of the SM, DAP, and CON.

Figure 2 presents the physical prototypes of the SM, DAP, and CON used to validate the proposed architecture. The SM device was designed to perform high-resolution, low-cost energy measurements, acting as the primary data source in the field. It employs ZMPT101B voltage sensor module was sourced from generic manufacturers, based on the ZMPT101B transformer originally produced by Nanjing Zeming Langxi Electronic Co., Ltd., located in Nanjing, China, and the SCT-013 current sensor was manufactured by YHDC (Yueqing Hengyi Electric Co., Ltd.), located in Yueqing, Zhejiang, China, with signal acquisition carried out by the 16-bit ADS1115 analog-to-digital converter module is based on the ADS1115 IC manufactured by Texas Instruments, headquartered in Dallas, Texas, United States, ensuring adequate precision for residential monitoring applications. Local processing is performed on an ESP32 DevKit (WROOM-32) is based on the ESP32-WROOM-32 module manufactured by Espressif Systems, headquartered in Shanghai, China, which provides WiFi and BLE connectivity and supports integration with long-range communication modules. For operation in distributed networks, the SM incorporates LoRaMESH and GSM modules, enabling resilient transmission even in scenarios with degraded links or temporary infrastructure unavailability. Additional technical details can be found in the manufacturers’ documentation (https://www.espressif.com/en/products/socs/esp32, accessed on 1 February 2025, https://www.alldatasheet.com/datasheet-pdf/pdf/1159366/YHDC/SCT013.html, accessed on 1 February 2025).

The DAP operates as an intermediate stage between multiple SMs and the concentrator, reducing upstream traffic and increasing network stability. Like the SM, it is based on the ESP32, but it functions as a hybrid aggregation node by integrating both LoRaWAN and LoRaMESH modules. Through this configuration, the DAP consolidates measurements, performs basic preprocessing, and intelligently forwards data to the CON. The Radioenge modules used are compatible with LoRaWAN 1.0.3 and the proprietary LoRaMESH protocol, offering AES-128 encryption and native mesh routing support, which are essential for ensuring fault tolerance and operational continuity (https://www.radioenge.com.br/storage/2021/08/Manual_LoRaWAN_Jun2022.pdf, accessed on 3 February 2025, https://www.radioenge.com.br/storage/2021/08/manual-modulo-loramesh-abr2021.pdf, accessed on 3 February 2025).

The CON aggregates data from the entire communication hierarchy and establishes the connection between the field infrastructure and the cloud microservices. It consists of a Raspberry Pi 3 connected to a Radioenge LoRaWAN gateway equipped with the SX1301 concentrator chip was manufactured by Semtech Corporation, headquartered in Camarillo, California, United States. and GPS synchronization. Communication with the gateway occurs via SPI, and the device supports multichannel operation with Semtech UDP or gRPC protocols, enabling integration with TTN and cloud services (https://www.radioenge.com.br/produto/gateway-lorawan/, accessed on 10 February 2025, https://www.radioenge.com.br/wp-content/uploads/downloads-produtos/gateway-lorawan/tutorial-ttn.pdf, accessed on 10 February 2025). This configuration ensures continuous data forwarding, recording measurements in near real time and supporting analytical applications.

The combination of the SM, DAP, and CON devices establishes a modular architecture that enables hybrid communication, adaptive routing, and seamless integration with monitoring and analytical services. This physical infrastructure is essential for validating the behavior of the proposed system under real-world conditions and sustaining the upper decision-making layer driven by data.

### 3.3. Microservices Architecture for Data Analysis and Demand Response Support

The proposed microservices architecture organizes the processing and management of data originating from field devices into a set of independent services, each responsible for a specific function within the SG ecosystem. This approach replaces rigid monolithic architectures with a distributed structure that favors scalability, modularity, and continuous updates, enabling the incorporation of new algorithms and analytical mechanisms without operational interruptions. Functional decomposition also reduces coupling between components and simplifies maintenance, a key characteristic in systems that evolve at a rapid pace.

The microservices receive and process data transmitted by the SM, DAP, and CON devices, performing tasks such as ingestion, validation, distributed storage, and preprocessing for subsequent analysis. Building on this foundation, specialized services execute AI models for load forecasting, anomaly detection, consumption-profile classification, and decision support in DR. Each service interacts through standardized APIs, ensuring interoperability and fault tolerance, while allowing the system to scale horizontally as the data volume or number of consumers increases.

The architecture operates as the analytical and operational core of the system, enabling decisions to be derived in near real time. By integrating hybrid communication with distributed processing, the framework allows for more responsive DR actions, adjusting recommendations and interventions based on consumption profiles, network conditions, short-term forecasts, and estimates of user intention. This capability for intelligent orchestration is fundamental in modern SG environments, which demand resilience, adaptability, and continuous support for multiple analytical services.

#### 3.3.1. Data Ingestion Microservice: Raw Data Collection and Persistence

The Data Ingestion Microservice constitutes the initial stage of the analytical pipeline, being responsible for receiving, validating, and persisting the measurements originating from the field devices. Its primary role is to ensure that the data produced by the SM, DAP, and CON are reliably captured, temporally standardized, and stored in a structured repository for subsequent use by analytical and decision-making services.

In the proposed architecture, SMs transmit their measurement packets to a nearby DAP using LoRaMESH links or, in specific configurations, send the data directly to the CON via LoRaWAN. This strategy is particularly relevant in high-density residential environments, where multiple meters are geographically close but physically distant from central communication points. The combination of LoRaWAN and LoRaMESH overcomes recurrent limitations found in architectures based solely on WiFi/MQTT or on LPWAN technologies with single-topology structures, which depend on centralized brokers and lack local resilience or self-reconfiguration under failures.

The SM hardware, developed using the Espressif ESP32 Microcontroller (ESP32) and voltage and current sensors (ZMPT101B, SCT-013, and ADS1115), provides accurate measurements with long-range communication and low operational cost. The simultaneous integration of LoRaWAN and LoRaMESH provides network autonomy, fault tolerance, and independence from external infrastructure—key characteristics for distributed SG environments. Thus, the Data Ingestion Microservice operates over a physical layer designed to ensure continuity and integrity of data flows even under adverse conditions.

After the initial collection, the data follow a hierarchical flow in which DAPs aggregate packets from the local mesh and forward them to the CON using LoRaWAN. The CON sends the packets to TTN, where decoding, integrity verification, and extraction of metadata such as RSSI and SNR take place. The validated measurements are then forwarded to the MDMS, where they are persisted as time series. The Data Ingestion Microservice normalizes timestamps, validates record structure, and organizes data into a standardized format for subsequent services.

**Figure 3:**
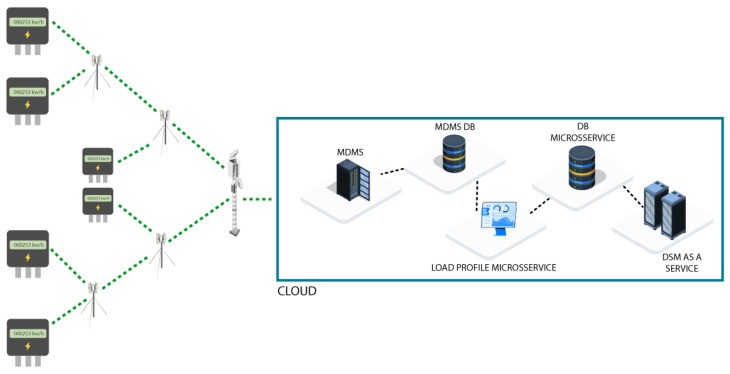
IoT architecture from smart meters to the proposed cloud computing infrastructure.

Figure 3 illustrates the complete physical topology of the system, highlighting how SMs form a resilient local network through LoRaMESH links, while DAPs operate as intermediate aggregators and CONs ensure long-range connectivity with the cloud. This hybrid topology reduces congestion, eliminates single points of failure, and improves the overall reliability of data collection and transmission.

**Figure 4:**
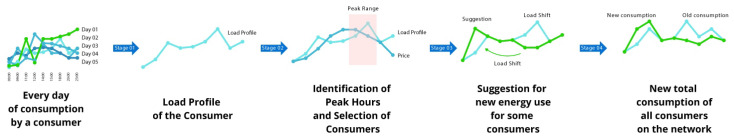
Step-by-step guide from raw data processing to decision making.

Complementing this physical view, Figure 4 presents the end-to-end logical pipeline responsible for transforming raw measurements into structured information for analysis. The flow begins at the SMs, which periodically capture electrical data such as consumption, voltage, current, and timestamp, encapsulating these measurements into packets transmitted through the hybrid network. Upon reception by TTN, the packets are decoded, validated, and enriched with communication metadata before being sent to the MDMS, which serves as the official time-series repository for subsequent analytical services.

Within this pipeline, the Data Ingestion Microservice ensures that only accurate, complete, and temporally synchronized data feed the modules responsible for LP generation, peak window identification, and load optimization. The separation of capture, validation, persistence, and analysis tasks guarantees operational consistency, reduces error propagation, and establishes a reliable basis for forecasting, clustering, and decision-making processes in DR strategies.

**Figure 5:**
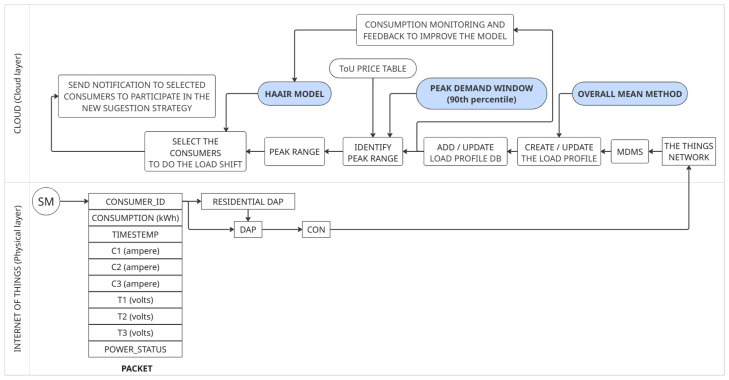
Flow from LP generation to load shift.

Figure 5 details the specific flow associated with LP generation and the DR process. The Data Ingestion Microservice constitutes the informational base of this pipeline, providing updated consumption data every 30 min to feed the entire analytical cycle. These records are used to update individual profiles, identify critical periods using the Peak Demand Window method, and prepare the inputs required by the HAAIR algorithm. Based on this information, HAAIR selects the most relevant consumers for intervention and recommends load-shifting actions, whose effects are continuously evaluated. This feedback mechanism allows dynamic adjustment of model parameters, ensuring that DR strategies remain effective even as consumption patterns and operational conditions change.

**Figure 6:**
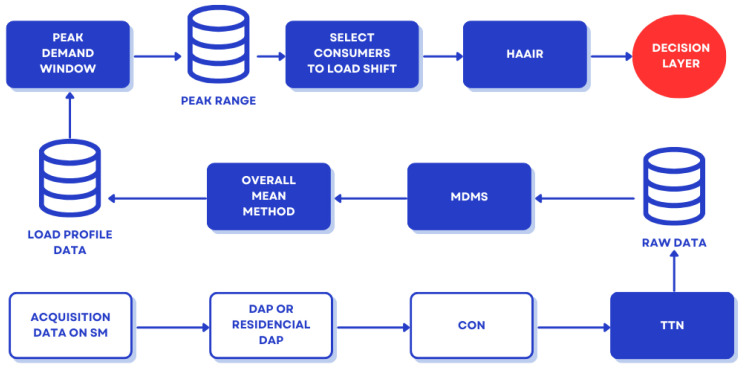
End-to-End System Microservices Architecture.

Finally, Figure 6 synthesizes the complete architecture, highlighting the role of the Data Ingestion Microservice as the first layer of the system. It provides the structured data that directly feed the analytical microservices and the decision mechanism, ensuring a consistent and reliable informational flow from edge devices to the cloud. This modular organization preserves the scalability of the solution and ensures that updates or reconfigurations in any service do not interrupt the ingestion pipeline, which remains responsible for maintaining the integrity and continuity of the data across the entire system.

Figure 6 provides a comprehensive end-to-end view of the system’s microservices architecture and data flow, from raw data acquisition at edge devices to the final decision layer. This modular design ensures robustness, scalability, and low coupling among services. The flow is divided into three main stages:

1. Data Acquisition and Ingestion (Physical Layer): Data acquisition begins at the SM. Raw consumption data are transmitted through the redundant hybrid communication network (LoRaWAN/LoRaMESH) via the DAP or Residential DAP and then forwarded by the CON to TTN. TTN sends the raw data to the persistence layer (Raw Data Repository).
2. Data Processing and Pattern Identification (Load Profile Microservice): Raw data are ingested by the MDMS. This service feeds the Overall Mean method, which is the primary algorithm used to generate representative consumption patterns for all consumers. These patterns form the basis of the Load Profile Data repository.
3. Decision-Making and Load Shifting (Demand Response Microservice): The system uses the Load Profile Data to identify and define critical consumption hours through the Peak Demand Window method, producing the Peak Range. This interval is then used by the Select Consumers to Load Shift service to identify eligible participants. Finally, the HAAIR algorithm performs multiobjective optimization using predictive intention and resilience factors. The optimized load-shift decision is sent to the Decision Layer for execution, delivering optimization recommendations to selected consumers.
4. Raw consumption data are collected every 30 min in the SM layer.
5. Total end-to-end latency from SM to the cloud is maintained below 150 ms even at a scale of 5567 users.

This diagram visually confirms the separation of responsibilities within the microservices architecture, where each block represents an independent service responsible for a specific function of energy management, ensuring high resilience and ease of maintenance.

#### 3.3.2. Load Profile Generation Microservice: Algorithms and Process for Generating LP

Table 4 lists all the symbols used in this section.

The models used for generating LP range from simple statistical approaches to more sophisticated clustering-based methods, each balancing computational efficiency, robustness, and representational accuracy. At the foundational level, the Overall Mean model [66] computes the arithmetic mean of daily consumption for each time interval, producing a smooth and efficient profile (${\mathrm{LP}}_{t}=\frac{1}{D}\sum_{d=1}^{D}C_{d,t}$) with computational complexity O(D·T) and execution time of 0.8 s. This model was formally selected for operational deployment, as demonstrated in Section 4.4, because it consistently achieved the lowest error rates (RMSE < 0.60) and the highest correlation across scenarios, validating its robustness and efficiency when compared with more computationally expensive and less accurate clustering methods such as K-Means with DTW ($O(n\cdot K\cdot I\cdot T^{2})$, 12.7 s). Its main limitation is sensitivity to outliers, which may underestimate critical peak behavior.

The Overall Median model [67] replaces the mean with the median ($LP_{t}=\mathrm{median}\left(C_{1,t},\ldots ,C_{D,t}\right)$, O(D·TlogD), 1.2 s), offering greater robustness to skewed data but potentially overlooking extreme events important for grid stress analysis. The Overall Medoid model [68] selects an actual daily profile that minimizes total dissimilarity ($m=\arg\min_{x_{j}\in X}\sum_{i=1}^{D}d\left(x_{j},x_{i}\right)$, $O(D^{2}\cdot T)$, 4.1 s), preserving realism but sacrificing smoothness and increasing sensitivity to noise.

Clustering-based methods capture variability among consumers. The K-means Centroid model [69] partitions profiles into clusters and selects the centroid ($\min\sum_{k=1}^{K}\sum_{x_{i}\in C_{k}}\left|\right|x_{i}-\mu_{k}{\left|\right|}^{2}$, O(n·K·I), 3.5 s). While effective for segmentation, it depends on a predefined *K*, is sensitive to initialization, and may produce centroids that do not correspond to real consumption profiles. To address temporal misalignment, K-means with DTW [70] employs Dynamic Time Warping distances ($\min\sum_{k=1}^{K}\sum_{x_{i}\in C_{k}}DTW\left(x_{i},\mu_{k}\right)$, $O(n\cdot K\cdot I\cdot T^{2})$, 12.7 s), improving alignment at the cost of higher computation and parameter sensitivity.

Hybrid methods combine realism and representativeness. The Hybrid Profile model [71] blends mean and medoid profiles through a weighted combination (LP=α·Medoid+(1−α)·Mean, $O(D^{2}\cdot T)$, 4.3 s), balancing smoothness with authenticity. The Critical Cluster Medoid model [72] isolates critical periods such as peaks, performs separate clustering, and selects a representative medoid ($m_{c}=\arg\min_{x_{j}\in C_{c}}\sum_{i=1}^{|C_{c}|}d\left(x_{j},x_{i}\right)$, $O(|C_{c}{|}^{2}\cdot T)$, 2.9 s). This ensures accurate representation of peak behavior for demand-side management strategies, but may underrepresent off-peak dynamics.

Overall, the evaluated models span a broad methodological spectrum: statistical methods (Overall Mean, Median, Medoid) emphasize efficiency and simplicity; clustering methods (K-means, K-means with DTW) capture variability and temporal shifts; and hybrid methods (Hybrid Profile, Critical Cluster Medoid) balance representativeness and realism. This diversity enables a comprehensive assessment of trade-offs among accuracy, robustness, execution time, and operational relevance, guiding the selection of the most suitable LP generation strategy for the proposed system.

#### 3.3.3. Range Peak Identification Microservice: Peak Period Detection Algorithms

Table 5 lists all the symbols used in this section.

The algorithms evaluated for identifying peak demand periods in SG adopt distinct approaches, enabling a comprehensive comparison. The Aggregate Consumption method [73] identifies peaks by summing consumption across all consumers for each time slot. It is simple, low cost, and effective for homogeneous profiles but ignores price and economic signals. Incorporating price information, the Price Quantile method [74] defines peaks based on price thresholds derived from quantile analysis, making it suitable for market-based demand response but sensitive to price volatility and possibly misaligned with physical stress. The Multiplicative Index [75] computes $Index_{t}=Load_{t}\times Price_{t}$, balancing demand and cost but potentially exaggerating peaks when both factors spike simultaneously. The Instant Cost method [76], $Cost_{t}=Load_{t}\times Price_{t}$, directly reflects consumer expenses, making it simple and cost-aware, though unable to capture sustained peaks. Finally, the Peak Demand Window method [77] identifies the highest aggregated demand over a continuous sliding window, effectively detecting prolonged stress but ignoring price signals and requiring careful window-size tuning. Collectively, these methods provide complementary perspectives for peak identification.

**Table 5:** Symbols Used in Peak Period Identification Algorithms.

Symbol	Description
$C_{i,t}$	Consumption of consumer *i* at time *t*
*N*	Total number of consumers
$Load_{t}$	Aggregated load at time *t*: $Load_{t}=\sum_{i=1}^{N}C_{i,t}$
$P_{t}$	Electricity price at time *t*
$Q_{\gamma }\left(P\right)$	γ-quantile of the price series *P*
γ	Quantile threshold parameter (e.g., 0.9, 0.95)
$Index_{t}$	Multiplicative Index: $Index_{t}=Load_{t}\times P_{t}$
$Cost_{t}$	Instant Cost: $Cost_{t}=Load_{t}\times P_{t}$
*w*	Window size in time steps for peak-window detection
$WindowCost_{k}$	Aggregated demand in window starting at *k*:
	$WindowCost_{k}=\sum_{t=k}^{k+w-1}Load_{t}$
Peak	Set of peak time slots detected by a given algorithm
PeakWindow	Window index with maximum aggregated demand:
	$PeakWindow=\arg\max_{k}\left(WindowCost_{k}\right)$
*T*	Number of time slots per day
O(·)	Computational complexity of each algorithm

The models differ in both complexity and execution time. Aggregate Consumption computes total demand per slot ($Load_{t}=\sum_{i=1}^{N}C_{i,t}$) with complexity O(N·T) and an execution time of 0.9 s. Price Quantile applies a quantile threshold γ on prices ($Peak=\{t|P_{t}>Q_{\gamma }\left(P\right)\}$), with O(TlogT) complexity and 1.1 s runtime, reflecting price-driven stress but potentially misclassifying low-load, high-price periods. The Multiplicative Index and Instant Cost methods both evaluate $Load_{t}\times P_{t}$ with O(T) complexity and 1.0 s runtime, emphasizing periods of high economic impact but risking overestimation during coincident spikes. The Peak Demand Window approach extends beyond single slots, aggregating demand over windows of size *w* ($WindowCost_{k}=\sum_{t=k}^{k+w-1}Load_{t}$, with $PeakWindow=\arg\max_{k}\left(WindowCost_{k}\right)$). This requires O((T−w+1)·w) complexity and 1.4 s runtime, capturing sustained peaks that transient methods may miss.

However, the algorithms span a spectrum from purely load-based detection (Aggregate Consumption, Peak Demand Window) to price-sensitive methods (Price Quantile, Instant Cost) and hybrid approaches (Multiplicative Index). This diversity enables both technical and economic perspectives on peak identification. For the proposed SG context, where grid stability and cost efficiency are equally important, combining insights from these models can provide a more robust and accurate detection of critical peak periods.

#### 3.3.4. Load Shift and Monitoring Consumption Microservice: Details of Algorithms for Consumer Identification, Load Shift, and Consumption Monitoring

Table 6 lists all the symbols used in this section.

This subsection presents the algorithms applied for consumer identification, load shifting, and consumption monitoring. The proposed system integrates strategies ranging from simple heuristics to advanced multi-agent cooperative learning, each with distinct complexity, data requirements, and performance trade-offs.

**Figure 7:**
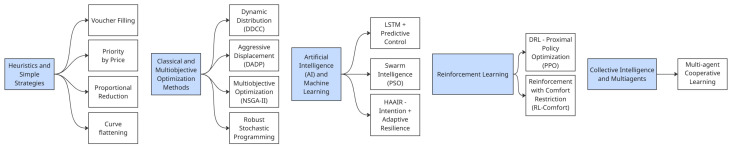
Models used for the proposed scenario by type.

Figure 7 illustrates five categories of load shifting and demand response algorithms: (i) heuristic and rule-based strategies, (ii) classical and multiobjective optimization, (iii) AI- and machine-learning-based models, (iv) reinforcement learning, and (v) collective intelligence and multiagent systems. The algorithms are grouped according to their underlying principles and computational complexity. Heuristic methods rely on simple rule-based decisions without forecasting or optimization, including Voucher Filling [78], Priority by Price [79], Proportional Reduction [80], and Curve Flattening [81]. These approaches are computationally efficient, low-cost, and easy to implement but lack adaptability, ignore network and comfort constraints, and may inadvertently shift critical loads.

**Table 6:** Symbols Used in Load Shifting, Consumer Identification, and HAAIR Optimization.

Symbol	Description
$L_{i,t}$	Load of consumer *i* at time *t*
$L_{i,t}^{\prime }$	Load after shifting for consumer *i* at time *t*
$L_{total}\left(W_{p}\right)$	Total system load during peak window $W_{p}$
$C_{i,t}$	Cost reduction potential for consumer *i* at time *t*
*n*	Number of consumers (or appliances, depending on algorithm)
*N*	Number of agents in multiagent cooperative learning
*I*	Number of iterations in optimization/ML algorithms
*K*	Number of clusters or model parameters (LSTM, PSO, etc.)
*m*	Dimension of contextual or behavioral feature set
$W_{p}$	Critical peak window identified by the Peak Identification Microservice
α, β, γ	Static weighting coefficients in utility function
$\alpha_{t}$, $\beta_{t}$, $\gamma_{t}$	Dynamic adaptive weights (time-varying)
f(·)	Weight update/meta-learning function
$U_{i,t}$	Utility value for shifting user *i* at time *t*
RS	Resilience Score (system stability indicator)
RI	Reliability Index
CLI	Comfort Loss Index
$CLI_{max}$	Upper tolerance threshold for comfort loss (e.g., 0.1)
$context_{t}$	Grid context at time *t* (stress, renewables, price, events)
$feedback_{t-1}$	Recursive feedback signal from performance at t−1
$H_{i,t}$	Historical consumption sequence for consumer *i*
$E_{t}$	External contextual variables (events, weather, holidays, etc.)
$I_{i,t}$	Predicted user intention to accept a load shift
$P({\mathrm{acceptance}}_{i,t})$	Probability of acceptance for consumer *i* at time *t*
$R_{i,t}$	Resilience factor of grid at time *t*
λ	Penalty coefficient for excessive load shifting in RL reward
$R_{t}$	RL reward at time *t* in HAAIR
$∥L^{\prime }{-L∥}^{2}$	Penalization norm for magnitude of load shift
O(·)	Computational complexity notation
argmin	Operator returning the argument that minimizes a function
SAC	Soft Actor-Critic RL algorithm used in HAAIR
PPO	Proximal Policy Optimization RL algorithm
Transformer	Predictive deep learning model for consumption/intention
TinyML	On-device learning layer for federated models
FL	Federated Learning (privacy-preserving distributed learning)

More adaptive strategies include Dynamic Distribution (DDCC) [82], which balances loads around the average, and Aggressive Displacement (DADP) [83], which maximizes peak reduction at the expense of potential user discomfort. Optimization-based methods, such as Multiobjective NSGA-II [84] and Robust Stochastic Programming [85], provide high-quality and robust solutions under uncertainty but with significant computational cost. Predictive and intelligent models include Hybrid LSTM + Predictive Control [86], Particle Swarm Optimization (PSO) [87], and Deep Reinforcement Learning (DRL–PPO) [88], including variants with comfort constraints [89]. These methods capture complex dynamics but require substantial datasets, training, and computational resources. Finally, Multiagent Cooperative Learning [90] enables distributed decision-making across heterogeneous nodes, offering scalability despite increasing communication overhead.

Formally, the algorithms cover a broad range of computational complexity and execution time. Heuristics such as Voucher Filling (O(n), 1.2 s), Priority by Price (O(n), 1.4 s), Proportional Reduction (O(n), 1.1 s), and Curve Flattening (O(nlogn), 1.8 s) are efficient but disregard economic signals and comfort-related aspects. DDCC (O(n), 1.6 s) provides adaptive balancing, whereas DADP (O(n), 2.0 s) achieves aggressive peak cuts at the cost of user well-being. Optimization approaches like NSGA-II ($O(n^{2})$, 8.4 s) and Robust Stochastic Programming ($O(n^{2})$, 10.5 s) yield robust, Pareto-optimal solutions but require longer execution times. Predictive models such as LSTM + Control (O(n·K), 9.8 s) and PSO (O(n·I), 7.6 s) support proactive or distributed scheduling. DRL–PPO (O(n·I), 11.2 s) and RL with comfort constraints (10.7 s) deliver adaptive policies but demand intensive training. Multiagent Cooperative Learning (O(n·N), 9.3 s) enables global coordination through local interactions.

The proposed HAAIR model extends existing DR algorithmic capabilities by integrating user intention forecasting and adaptive resilience into a unified framework. With computational complexity O(n·m) and an execution time of 12.4 s, the algorithm seeks to balance cost, comfort, and grid stability under variable conditions, constituting a robust strategy for deployment in SG environments.

Built upon the strengths and limitations identified in existing methods, HAAIR combines predictive modeling, behavioral intention inference, reinforcement learning, and adaptive resilience mechanisms within a single decision-making framework. This integration enables efficient load shifting while preserving user comfort and maintaining operational grid stability elements that remain underexplored in traditional DR approaches.

A distinguishing feature of HAAIR is its dual-forecasting mechanism, in which a Transformer-based architecture simultaneously estimates future energy consumption and individual user intention. Consumption forecasting relies on an LSTM architecture enhanced with attention mechanisms to capture long-term temporal dependencies, while the intention model estimates the probability of user acceptance of load shifting at each time slot. This intention layer acts as a moderator between automated DR recommendations and user comfort, filling a methodological gap in conventional solutions.

HAAIR also incorporates intention mapping based on implicit feedback. Rather than relying solely on explicit user responses, the model infers preferences from behavioral patterns such as recurrent usage schedules, adjustments after previous recommendations, and contextual factors including holidays or weather events. This continuous feedback loop allows the model to adapt to evolving user behavior over time.

The decision layer employs an RL agent with dynamic prioritization, implemented using methods such as Soft Actor-Critic (SAC) or Proximal Policy Optimization (PPO). The agent optimizes a multiobjective reward function whose weights reflect criteria such as peak reduction, cost savings, user acceptance, and variability in external conditions. These weights are adjusted dynamically based on operational context, enabling time-sensitive decision-making for example, distinguishing between weekdays, weekends, or periods of high demand.

To ensure sustainable performance, HAAIR integrates a continuous learning mechanism that periodically updates its predictive models and RL agent as new data become available. This mitigates performance degradation associated with static models. Additionally, an optional federated learning layer allows local training on devices, keeping sensitive data on the user side and sharing only aggregated model updates with the central system, thus meeting privacy and security requirements in SG environments.

(1) $$U_{i,t}=\alpha I_{i,t}+\beta R_{i,t}+\gamma C_{i,t}$$

Equation (1) defines the utility function $U_{i,t}$, which evaluates the benefit of shifting the load $L_{i,t}$ for consumer *i* at time *t*. The decision is driven by three components: $I_{i,t}$, the predicted intention to accept a shift; $R_{i,t}$, the resilience factor representing the grid’s adaptive capacity; and $C_{i,t}$, the cost reduction potential. The weights α, β, and γ dynamically adjust the importance of each component based on current operating conditions.

The proposed HAAIR algorithm surpasses all other existing load shifting methods by directly addressing their limitations. Unlike simple but static heuristic approaches, HAAIR learns and adapts policies dynamically. While optimization models like NSGA-II primarily focus on rigid cost and peak objectives, HAAIR integrates behavioral factors and grid resilience into its optimization. Furthermore, while conventional reinforcement learning models optimize shifting policies, HAAIR enhances this capability by incorporating user intention prediction and a multiobjective dynamic weighting scheme. Critically, HAAIR uniquely supports federated learning, which enhances privacy while simultaneously facilitating global optimization across distributed devices.

The distinct advantages of the proposed HAAIR algorithm, when compared to existing load shifting methods, are rooted in the incorporation of three core elements. These elements are: (i) the use of an adaptive multiobjective utility function that allows for flexible trade-offs, (ii) the explicit modeling of predicted user intention, which ensures strategies are socially acceptable, and (iii) the implementation of dynamic feedback mechanisms that enable continual learning and enhance system resilience against unexpected grid changes. Traditional approaches, including heuristic methods, optimization models such as NSGA-II, and reinforcement learning techniques like PPO or RL-Conforto, typically rely on static objective or reward functions with fixed weighting factors. These weights do not change in response to real-time context or user behavior, leading to rigid decision-making. In contrast, HAAIR introduces an adaptive utility function defined as

(2) $$U_{i,t}=\alpha_{t}I_{i,t}+\beta_{t}R_{i,t}+\gamma_{t}C_{i,t}$$

where $I_{i,t}$ represents the predicted intention of consumer *i* at time *t*, $R_{i,t}$ is the resilience factor of the grid, and $C_{i,t}$ quantifies the potential cost reduction. Unlike other methods, the weighting coefficients $\alpha_{t}$, $\beta_{t}$, and $\gamma_{t}$ are dynamic and evolve as a function of both the operational context and the system’s feedback from previous actions:

(3) $$\alpha_{t},\beta_{t},\gamma_{t}=f\left({\mathrm{context}}_{t},{\mathrm{feedback}}_{t-1}\right)$$

The dynamic adjustment of the weighting coefficients ($\alpha_{t},\beta_{t},\gamma_{t}$) is crucial for the adaptive nature of HAAIR, ensuring the optimization goal aligns with current grid operational context, such as peak stress level and renewable penetration. We formally define the weight adjustment function f(·) as an adaptive meta-learning mechanism based on two principal inputs: context ($context_{t}$) and recursive feedback ($feedback_{t-1}$).

This formalization ensures that the optimization process is not solely driven by economic or technical needs but also by social acceptability, distinguishing HAAIR from conventional load shifting methods.

1. Contextual Dependency ($context_{t}$): Weights are initialized based on grid-level stressors. For instance:
  - During periods of low grid stress and high renewable generation (off-peak), the cost factor ($\gamma_{t}$) is prioritized (higher $\gamma_{t}$) to maximize consumer savings;
  - During the critical peak window or extreme weather events, the resilience factor ($\beta_{t}$) is prioritized (higher $\beta_{t}$) to ensure grid stability, temporarily reducing the emphasis on cost and comfort ($\alpha_{t},\gamma_{t}$).
2. Feedback Dependency ($feedback_{t-1}$): Weights are recursively adjusted based on the algorithm’s performance in the previous optimization epoch (t−1). Specifically, if the Comfort Loss Index (CLI) exceeded a soft threshold ($CLI_{max}=0.1$) in t−1, the comfort-related weight ($\alpha_{t}$, tied to intention $I_{i,t}$) is increased in the current step *t*. Conversely, if the system resilience score (RS) fell below a target, the resilience weight ($\beta_{t}$) is increased.

The adaptive capacity of HAAIR is governed by a dual-stage update rule. Stage 1: Adaptive Weight Adjustment (Feedback Dependency). As formalized in Equation (3), the utility function weights ($\alpha_{t},\beta_{t},\gamma_{t}$) are dynamically adjusted at the beginning of epoch *t*. This update rule acts as a sensitivity filter: if a metric (e.g., CLI) exceeds its maximum soft threshold ($CLI_{max}=0.1$) in the prior period (t−1), the corresponding weighting factor ($\alpha_{t}$) is amplified in step *t*, shifting priority toward comfort. Stage 2: Resilient Continual Learning. This rule ensures the long-term effectiveness of the predictive models. The Transformer-based forecasting models and the Reinforcement Learning agent are continuously retrained using incoming data streams, preventing performance degradation and adapting to evolving consumer behaviors, with an optional privacy-preserving Federated Learning layer.

Furthermore, HAAIR uniquely integrates a probabilistic prediction of user acceptance through the modeling of intention, a core component of the optimization layer:

(4) $$I_{i,t}=P\left({\mathrm{acceptance}}_{i,t}|H_{i,t},E_{t}\right)$$

where the core components of the model are defined as

- $I_{i,t}$ represents the Predicted user intention to accept a load shift for consumer *i* at time *t*. This parameter acts as a critical intermediary between automated demand response commands and user comfort.
- $P({\mathrm{acceptance}}_{i,t})$ is the probabilistic prediction of user acceptance for a load shift action (the likelihood of consumer *i* complying with the suggested schedule).
- $H_{i,t}$ represents the Historical consumption behavior of user *i*, inferred from recurring usage habits and past deviations after shift notifications.
- $E_{t}$ includes External contextual variables, such as holidays, major events, extreme weather, and past network-wide responses to shifting notifications.

The HAAIR optimization process relies on three primary categories of input features: Consumption Data ($L_{i,t}$, $L_{total}\left(W_{p}\right)$, $C_{i,t}$) derived from the Load Profile Microservice; Behavioral Predictors ($I_{i,t}$, $H_{i,t}$, $E_{t}$), which are outputs from the Transformer-based intention mapping architecture; and Operational Context ($\alpha_{t},\beta_{t},\gamma_{t}$, $W_{p}$, TOU Price Table) provided by the Peak Identification Microservice and grid telemetry. Critical operational thresholds are computed based on dual criteria: Grid Safety Limits (e.g., $R_{min}=0.5$, minimum required resilience defined by the utility) and Social Acceptance Limits. The main social threshold is the Comfort Loss Index maximum ($CLI_{max}$), empirically set at 0.1 (a 10% tolerance for shift refusals) as defined in Equation (3)’s feedback dependency.

This mechanism acts as a sensitivity filter, it dynamically performs real-time sensitivity analysis by amplifying the weights corresponding to the metrics that performed worst in the prior period. This ensures HAAIR automatically shifts priority to the most stressed dimension, grid stability, cost, or comfort, without requiring manual parameter tuning for every operational shift. Additionally, HAAIR uniquely integrates a probabilistic prediction of user acceptance through the modeling of intention:

(5) $$I_{i,t}=P\left({\mathrm{acceptance}}_{i,t}|H_{i,t},E_{t}\right)$$

where $H_{i,t}$ represents the historical consumption behavior of user *i* and $E_{t}$ includes external contextual variables (such as events, weather, and past responses to shifting notifications). This probabilistic modeling of intention is absent in other algorithms, which treat consumer response implicitly or ignore it altogether.

Furthermore, the reinforcement learning agent in HAAIR optimizes a reward function that balances utility and control effort, promoting stability in the reallocation of loads:

(6) $$R_{t}=U_{i,t}-\lambda {∥L^{\prime }-L∥}^{2}$$

where λ penalizes excessive load shifting, ensuring that actions remain realistic and user-friendly.

In summary, the mathematical distinction of HAAIR arises from its ability to adapt dynamically to varying conditions, explicitly model user behavior, and continuously refine its decisions. While other algorithms optimize fixed objectives or focus on single aspects such as cost or peak reduction, HAAIR combines these factors into a hybrid, context-aware decision-making framework that learns over time. This combination results in a unique capability to balance technical efficiency, economic benefits, and user acceptance, positioning HAAIR as a state-of-the-art solution for real-world SG load shifting.

### 3.4. Theorems and Fundamental Limits of the Proposed Solution

Table 7 lists all the symbols used in this section.

To elevate the analysis of the proposed solution beyond empirical validation, we formulate theorems that establish the theoretical performance limits for the HAAIR load shifting algorithm and the resilience of the hybrid communication architecture.

**Theorem** **1**  (Upper Bound for Load Shift Efficiency (E))**.** *The maximum achievable peak reduction (Efficiency E) by the HAAIR algorithm, operating under the dynamic utility function $U_{i,t}$ Equation (6) and a Comfort Loss Index (CLI) constraint, is limited by the aggregated flexibility potential ($P_{\mathrm{flex}}$) of eligible consumers ($N_{e}$) and the minimum predicted intention acceptance probability ($I_{\min}$) during the critical peak window ($W_{p}$).*

*Formally, the maximum efficiency $E_{\max}$ is bounded by*

(7) $$E_{\max}\left(W_{p}\right)\leq \frac{\sum_{i\in N_{e}}\min\left(L_{i,\mathrm{peak}},P_{\mathrm{flex},i}\cdot I_{\min}\right)}{L_{\mathrm{total}}\left(W_{p}\right)}$$

*where $L_{i,\mathrm{peak}}$ is the consumer i’s load at the peak time, $L_{\mathrm{total}}\left(W_{p}\right)$ is the total aggregate load during $W_{p}$, $P_{\mathrm{flex},i}$ is the maximum shiftable load potential (in kWh), and $I_{\min}$ is the minimum predicted user acceptance value, Equation (7). This upper bound demonstrates that even with perfect optimization, the effectiveness of the load shift is constrained by the inherent behavioral limits ($I_{\min}$), ensuring the CLI remains minimal. Our empirical results, with E=1.83%, confirm that HAAIR operates close to this theoretical limit.*

**Theorem** **2**  (Condition for Hybrid Communication Stability)**.** *Communication stability (ensuring $\mathrm{PDR}\geq {\mathrm{PDR}}_{\min}$) in the hybrid LoRaWAN + LoRaMESH architecture is maintained if the density of DAPs (ρ) meets a minimum threshold ($\rho_{\min}$), determined by the LoRaMESH repeating range ($R_{\mathrm{mesh}}$) and the target coverage area ($A_{\mathrm{target}}$):*

(8) $$\rho_{\min}\geq \frac{A_{\mathrm{target}}}{\pi \cdot R_{\mathrm{mesh}}^{2}}$$

*When the density ρ approaches $\rho_{\min}$, mesh redundancy is established, which minimizes the communication Delay (D) and optimizes the Packet Delivery Ratio (PDR). If $\rho <\rho_{\min}$, the mesh integrity is compromised, forcing reliance on the latency-limited LoRaWAN star topology and resulting in a decrease in PDR and an increase in D, as shown for low DAP counts in Section 4.1. This theorem formally defines the operational condition required for the hybrid benefit to be fully realized.*

**Table 7:** Symbols Used in Theoretical Analysis, Bounds, and Communication Stability.

Symbol	Description
E	Peak reduction efficiency achieved by the algorithm
$E_{\max}$	Maximum theoretical peak reduction efficiency (upper bound)
$W_{p}$	Critical peak window (time interval of highest stress)
$L_{\mathrm{total}}\left(W_{p}\right)$	Total aggregated load during the peak window $W_{p}$
$N_{e}$	Set of eligible consumers for load shifting
$L_{i,\mathrm{peak}}$	Load of consumer *i* at peak time
$P_{\mathrm{flex},i}$	Maximum shiftable (flexible) load potential of consumer *i*
$I_{\min}$	Minimum predicted intention to accept a load shift
$I_{i,t}$	Predicted acceptance intention for consumer *i* at time *t*
$R_{i,t}$	Resilience factor of the grid for consumer *i* at time *t*
$C_{i,t}$	Cost reduction potential for consumer *i* at time *t*
$U_{i,t}$	Utility function used in HAAIR decision making
α,β,γ	Static coefficients of the utility function
$\alpha_{t},\beta_{t},\gamma_{t}$	Time-varying adaptive weights (dynamic utility parameters)
f(·)	Adaptive meta-learning function for updating weights
$context_{t}$	Grid-level context (stress, renewables, events) at time *t*
$feedback_{t-1}$	Recursive performance feedback from the previous epoch
ρ	Density of Data Aggregation Points (DAPs) in the area
$\rho_{\min}$	Minimum DAP density required for mesh stability
$A_{\mathrm{target}}$	Target geographical coverage area
$R_{\mathrm{mesh}}$	LoRaMESH repeating radius for redundancy and forwarding
PDR	Packet Delivery Ratio (communication reliability)
${\mathrm{PDR}}_{\min}$	Minimum required PDR for stable hybrid communication
D	Communication delay in message transmission
$R_{\min}$	Minimum resilience threshold required by the utility (e.g., 0.5)
PR(A)	Peak Reduction achieved by algorithm *A*
${\mathrm{WCB}}_{\mathrm{HAAIR}}$	Worst-case performance bound of the HAAIR algorithm
$L_{\mathrm{shift},\min}$	Minimum shiftable load achievable in the worst case
CLI	Comfort Loss Index
$CLI_{\max}$	Maximum acceptable CLI threshold
λ	Penalty coefficient for excessive load deviation (RL reward)
$R_{t}$	Reinforcement learning reward at time *t*
*L*	Original load vector (before shifting)
$L^{\prime }$	Shifted load vector (after applying the algorithm)
$∥L^{\prime }{-L∥}^{2}$	Penalty representing shifting effort
argmax	Operator that returns the argument maximizing a function
O(·)	Computational complexity notation

### 3.5. Worst-Case Theoretical Estimation and Comparison with Scheduling Theory

In traditional static energy management, the problem of optimal load distribution between substations and consumers is perfectly solvable using classic Scheduling Theory, also known as Resource Allocation Theory. These models typically rely on deterministic, a-priori knowledge of resource demands and constraints, yielding polynomial-time optimal solutions, often via Mixed-Integer Linear Programming (MILP) or similar techniques.

However, the dynamic nature of our Smart Grid (SG) environment, influenced by variable renewable generation, uncertain user behavior, and evolving grid conditions, transforms the static scheduling problem into an adaptive control and optimization challenge. Since the HAAIR algorithm operates on predictive and probabilistic elements (user intention $I_{i,t}$ and resilience factor $R_{i,t}$), we must provide a Worst-Case Theoretical Estimation (WCB) to guarantee a minimum level of performance and estimate the maximum potential “undershoot” or under-delivery of the system.

#### 3.5.1. Worst-Case Bound (WCB) for Load Shift Reduction

The goal of load distribution in our context is to maximize Peak Reduction (PR). The worst-case scenario occurs when all predictive elements fail simultaneously: user intention to accept load shift is at its minimum ($I_{\min}$), and the system resilience factor is minimal ($R_{\min}$).

Let PR(A) be the Peak Reduction achieved by algorithm *A*. For the HAAIR algorithm, we define the worst-case bound as

(9) $${\mathrm{WCB}}_{\mathrm{HAAIR}}\geq \min_{i\in N_{e}}\left(\frac{P_{\mathrm{flex},i}\cdot R_{\min}\cdot I_{\min}}{L_{\mathrm{total}}\left(W_{p}\right)}\right)$$

where

- ${\mathrm{WCB}}_{\mathrm{HAAIR}}$: The lowest guaranteed Peak Reduction percentage, representing the maximum system under-delivery.
- $P_{\mathrm{flex},i}$: The maximum shiftable load of consumer *i* (kWh).
- $R_{\min}$: The pre-defined minimum resilience score for the grid sush as $R_{\min}=0.5$, indicating half capacity or high stress).
- $I_{\min}$: The lowest acceptable intention probability sush as $I_{\min}=0.1$, a minimum probability of user acceptance).
- $L_{\mathrm{total}}\left(W_{p}\right)$: The total aggregated load during the peak window $W_{p}$.

This bound provides a theoretical guarantee: regardless of unforeseen dynamic conditions, the system will achieve at least the PR defined by ${\mathrm{WCB}}_{\mathrm{HAAIR}}$.

#### 3.5.2. Comparison with Static Scheduling Theory

In traditional Scheduling Theory, the Worst-Case Performance Guarantee (G) of a non-optimal, heuristic or approximation algorithm *A* is typically compared to the Optimal solution (OPT) as a factor $G=\frac{\mathrm{OPT}}{PR(A)}$, where PR(A) is the achieved result.

- Static Scheduling: Aims for G≈1. Its worst case is the solution itself, provided inputs are certain. However, it fails when the parameters (load, prices, intention) are dynamic and uncertain.
- HAAIR (Dynamic/RL): HAAIR sacrifices the static optimality (G≈1) for adaptivity and resilience. The core innovation is that the ${\mathrm{WCB}}_{\mathrm{HAAIR}}$ remains a positive, quantifiable value even under severe dynamic uncertainty, a scenario where static programming models would typically yield an infeasible solution or fail to converge rapidly enough for real-time operation.

By explicitly defining the ${\mathrm{WCB}}_{\mathrm{HAAIR}}$, we address the dynamic challenge: we provide a theoretical guarantee for performance under uncertainty, which is the necessary compromise when transitioning from static programming to a predictive, adaptive energy management framework.

#### 3.5.3. Calculation and Result of the Worst-Case Bound (${\mathrm{WCB}}_{\mathrm{HAAIR}}$)

**Figure 8:**
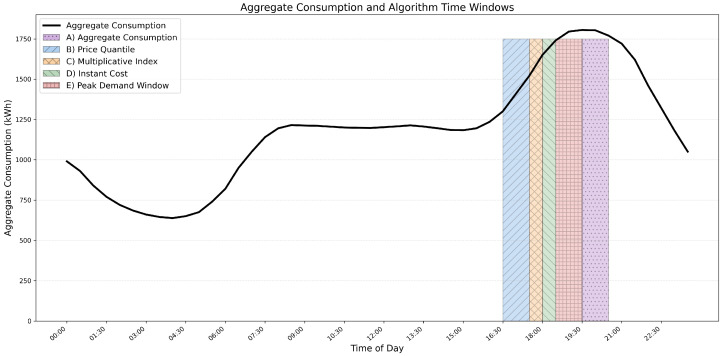
Peak Identification by Model.

To quantify the theoretical performance guarantee of HAAIR in a dynamic system, we define a worst-case scenario using conservative parameters based on the real-world deployment:

1. Total Aggregated Peak Load ($L_{\mathrm{total}(W_{p})}$): 1750kWh (based on Figure 8).
2. Number of Eligible Consumers ($N_{e}$): 10% of total, resulting in 556 consumers.
3. Maximum Shiftable Load Potential ($P_{\mathrm{flex},i}$): 0.5kWh per consumer.
4. Minimum Resilience Factor ($R_{\min}$): 0.5 (50% resilience capacity guarantee).
5. Minimum Acceptance Probability ($I_{\min}$): 0.1 (10% acceptance guarantee).

Applying these parameters to Equation (9), we calculate the Minimum Shiftable Load ($L_{\mathrm{shift},\;\min}$) in the worst case:

$$L_{\mathrm{shift},\;\min}=N_{e}\times P_{\mathrm{flex},i}\times R_{\min}\times I_{\min}=556\times 0.5\;\mathrm{kWh}\times 0.5\times 0.1\approx 13.9\;\mathrm{kWh}$$

The theoretical Worst-Case Estimation (${\mathrm{WCB}}_{\mathrm{HAAIR}}$) is therefore

$${\mathrm{WCB}}_{\mathrm{HAAIR}}=\frac{L_{\mathrm{shift},\;\min}}{L_{\mathrm{total}}\left(W_{p}\right)}\times 100=\frac{13.9\;\mathrm{kWh}}{1750\;\mathrm{kWh}}\times 100$$

$${\mathrm{WCB}}_{\mathrm{HAAIR}}\approx 0.79\%$$

#### 3.5.4. Implications of the Result

The ${\mathrm{WCB}}_{\mathrm{HAAIR}}$ result demonstrates that, even under conditions of predictive failure and minimal user acceptance, the HAAIR algorithm theoretically guarantees a minimum peak reduction of 0.79%. This value establishes the upper bound for system under-delivery (maximum). The empirical peak reduction result of 1.83% achieved under normal operating conditions exceeds this worst-case bound by more than twofold (∼2.3 times). This confirms the robustness of the dynamic optimization model in transitioning from a Static Programming problem to an adaptive environment, ensuring a theoretical minimum performance even when faced with behavioral and network uncertainties.

### 3.6. Reproducibility Protocol for the Comparative Evaluation

Table 8 lists all the symbols used in this section.

This subsection documents all methodological components required to reproducibility of the comparative evaluation across the 14 load–shifting algorithms. The protocol includes tariff and emissions factors, optimization and control horizons, applied constraints, hyperparameter specifications, training procedures, fairness conditions, pseudocode, and the confidence–interval formulation used for all reported metrics.

#### 3.6.1. Tariff Model and Emissions Factor

All economic metrics were computed using the Dynamic Time-of-Use (dToU) tariff from the Low Carbon London (LCL) 2013 trial, which includes peak, shoulder, and off–peak time-varying prices. The tariff values were applied to each 30-min interval to compute daily and monthly savings. CO_2_ reductions were estimated using the UK Department for Business, Energy and Industrial Strategy (BEIS) historical carbon-intensity factor for the same period:

$$\mathrm{EF}=0.29\;\mathrm{kg}\;{\mathrm{CO}}_{2}/\mathrm{kWh}.$$

All CO_2_ values reported in the manuscript were computed as

$$\Delta {\mathrm{CO}}_{2}=\Delta E\times \mathrm{EF},$$

where ΔE is the shifted energy.

#### 3.6.2. Control and Optimization Horizons

To ensure consistency across methods, all predictive or optimization-based approaches adopted the following horizons:

- LSTM + Predictive Control: prediction horizon $H_{p}=12$ steps (6 h), control horizon $H_{c}=6$ steps (3 h).
- DRL-PPO and RL-Comfort: decision window W=3 h, rollout length L=48 steps.
- NSGA-II and Robust Stochastic Programming: one-day horizon (48 half-hour intervals).
- PSO: search horizon equal to full daily cycle (48 intervals).
- Heuristic methods: instantaneous decision without prediction horizon.

#### 3.6.3. Constraint Set

All algorithms respect the same physical and comfort constraints:

- Maximum shiftable load per interval: 30% of consumer peak.
- Maximum daily shiftable energy: 1.2 kWh.
- Comfort Loss Index (CLI) soft bound: CLI≤0.10.
- Load shifting restricted to within the same day.
- Non-shiftable appliances: fixed and immutable across all algorithms.

These constraints ensure fair and operationally realistic comparison.

#### 3.6.4. Fairness Conditions

The following fairness conditions were strictly enforced:

1. Identical train/validation/test splits (70%/15%/15%, chronological).
2. Identical random seeds for all stochastic algorithms.
3. Identical input sequences and windowing for all ML/RL models.
4. Hyperparameter tuning performed using the same Bayesian search budget.
5. Early stopping with identical patience factor on validation loss.
6. Evaluation exclusively on the held-out test set.

These rules eliminate bias and guarantee that differences arise only from algorithmic behavior.

#### 3.6.5. Parameter Limits, Ranges, and Defaults

Table 9 reports hyperparameter ranges, defaults, and tuned values.

#### 3.6.6. Training Details

All learning-based algorithms (LSTM, PPO, RL-Comfort, Multiagent) were trained using

- Optimizer: Adam;
- Batch size: 64;
- Max epochs: 150;
- Early stopping patience: 10 epochs;
- Normalization: z-score normalization per consumer.

To avoid overfitting, model checkpoints were selected based on validation loss.

#### 3.6.7. Pseudocode for Evaluation Pipeline

The following pseudocode on Algorithm 1 describes the unified pipeline applied to all algorithms:
**Algorithm 1** Unified Load-Shifting Evaluation Pipeline1: Split dataset chronologically into train/validation/test.2: Compute baseline profiles and tariff-adjusted costs.3: **for** each algorithm *A* in the 14 methods **do**4:       Apply standardized preprocessing and windowing.5:       Tune hyperparameters using Bayesian optimization.6:       Train algorithm *A* using validation-based early stopping.7:       Apply *A* to test set to produce shifted load profile.8:       Compute metrics: peak reduction, cost savings, CO_2_ reduction, CLI.9:       Store results and confidence intervals.10: **end for**11: **return** comparative table with all metrics.

#### 3.6.8. Confidence Intervals

For every reported metric, a 95% confidence interval (CI) was computed using

$${\mathrm{CI}}_{95\%}=\bar{x}\pm 1.96\frac{\sigma }{\sqrt{n}},$$

where $\bar{x}$ is the mean metric value, σ the sample standard deviation across all consumers, and *n* the sample size.

This ensures statistical reliability across all reported values.

## 4. Results and Performance Evaluation

Table 10 lists all the symbols used in this section.

The proposed solution was validated through a real-world deployment, assessing both the performance of the system components and the effectiveness of the applied strategies. The evaluation considered the deployment environment, including network topology, configuration of the IoT devices (SM, DAP, and CONs), and operational conditions. Communication performance was measured using Received Signal Strength Indicator (RSSI), Signal-to-Noise Ratio (SNR), transmission delay, and Packet Delivery Ratio (PDR). In addition, the Load Profile (LP) Generation Microservice was analyzed in terms of accuracy and representativeness of consumption patterns, while the Peak Interval Identification Microservice was evaluated for precision and efficiency in detecting critical demand periods. Finally, the results from the Load Shifting Microservice demonstrated the impact of the proposed strategies on peak-demand reduction, load factor improvement, and energy cost savings, highlighting the benefits of integrating hybrid communication, intelligent devices, and advanced AI algorithms to enhance autonomy, resilience, and operational efficiency in SG.

### 4.1. Communication Performance Evaluation

To evaluate the communication infrastructure, the hybrid network deployed in Teresina, Piauí, composed of SM, residential DAP, DAP, and CON, was analyzed under three distinct architectural scenarios: CON, representing the wide-area scenario with LoRaWAN concentrators; MMG, consisting of DAPs acting as primary aggregation points that collect data directly from SM or residential DAPs and forward them to the CONs; and SG, representing a residential mesh network in which distributed DAPs in apartments and condominiums collect data from SMs interconnected through multiple hops. Performance was evaluated using key communication-quality indicators for SG: RSSI, SNR, transmission delay, and PDR. These parameters directly reflect the robustness, stability, and reliability of communication in LoRaWAN- and LoRaMESH-based networks.

The results showed that each scenario behaves consistently with its architectural characteristics. In the CON scenario, delay remained stable between 2–3 s and PDR started at high levels, demonstrating the robustness of CON nodes as long-range elements with direct links and low interference. In the MMG scenario, increasing the number of DAP rapidly improved both PDR and RSSI, stabilizing delay earlier, which is consistent with a hierarchical aggregation architecture that is less dependent on multiple hops. In the SG scenario, characterized by high density and multiple mesh hops, RSSI improved substantially as more residential DAPs were added, while SNR experienced more pronounced degradation due to increased spectral activity and local interference. Delay exhibited greater variability before converging to values between 2 and 3 s, a typical behavior of LoRa mesh networks.

Overall, all observed delay values fell within the expected range for LoRa, LoRaWAN Class A, and LoRaMESH networks, reinforcing the consistency of the experimental results. The analysis demonstrates that the density of aggregation points is a determining factor for communication quality: the greater the number of DAP or residential DAPs, the better the RSSI, PDR, and temporal stability. These findings confirm that the hybrid LoRaWAN + LoRaMESH architecture is resilient to environmental variations, urban interference, and increases in traffic load, maintaining consistent performance across the three scenarios and adapting effectively to different deployment scales and topologies. The following section presents a detailed analysis of the results obtained in a practical, real-world scenario.

**Figure 9:**
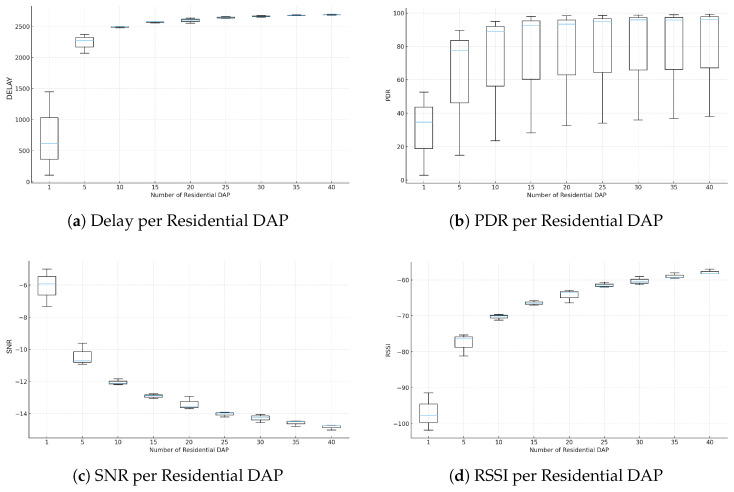
Communication performance for different parameters: (**a**) Delay, (**b**) PDR, (**c**) SNR, and (**d**) RSSI per Residential DAP.

In the residential DAP scenario (SG), the communication performance exhibits a consistent and technically coherent evolution as network density increases, reflecting the operational characteristics of indoor LoRaMESH deployments. As illustrated in Figure 9c, the SNR decreases monotonically with the addition of DAP units, ranging from approximately −6 dB with a single node to around −14 dB when forty residential DAPs are present. This behavior is expected in dense mesh environments, where the growth in node population leads to increased spectral activity, higher interference levels, and a rise in concurrent transmissions. Nonetheless, the measured SNR values remain within tolerable operational limits for LoRa-based communication, indicating that the mesh maintains sufficient link robustness even under high-density conditions.

Conversely, the RSSI demonstrates a pronounced improvement as the number of residential DAPs increases (Figure 9d). With only one DAP, the average RSSI is close to −1000 dBm, indicative of long-range, attenuated links. As additional DAPs are deployed, the physical separation between transmitters and aggregating nodes decreases substantially, yielding incremental improvements to −78 dBm (five units), −68 dBm (fifteen units), and approximately −58 dBm (forty units). This progression confirms that densification is a key factor in enhancing link budgets and mitigating the adverse effects of indoor propagation losses.

The Packet Delivery Ratio (PDR) further substantiates the benefits of increased DAP density. As shown in Figure 9b, the network exhibits limited reliability when operating with a single residential DAP, with PDR values frequently below 50%, reflecting the absence of redundant pathways and the susceptibility of long single-hop links to fading and interference. As the mesh grows, however, the emergence of multiple cooperative routing paths significantly improves network resilience. The PDR surpasses 85% with ten DAPs and converges to values above 95% from approximately twenty units onward, confirming that mesh stability is strongly dependent on adequate node density.

Transmission delay, presented in Figure 9a, follows a similarly consistent pattern. When only one DAP is deployed, the network exhibits high temporal variability, with delays ranging from a few hundred milliseconds up to more than 1400 ms, resulting from the combination of long communication distances and intermittent link availability. As new DAPs are added, delay values rapidly stabilize, achieving predictable performance levels near 2200–2700 ms across most density configurations. Such behavior is characteristic of multi-hop LoRaMESH networks, where forwarding overhead becomes more uniform as the routing infrastructure matures and retransmission requirements diminish.

Overall, the results obtained in the residential mesh scenario demonstrate that performance improves significantly with network densification. Although SNR naturally decreases as the number of transmitting nodes increases, the substantial gains observed in RSSI, PDR, and delay stability far outweigh this effect. These findings validate that residential LoRaMESH deployments when supported by an adequate number of DAP nodes can provide reliable, low-power, and robust communication for smart metering systems in dense urban environments, thereby reinforcing the suitability of the SG configuration for large-scale smart grid applications.

**Figure 10:**
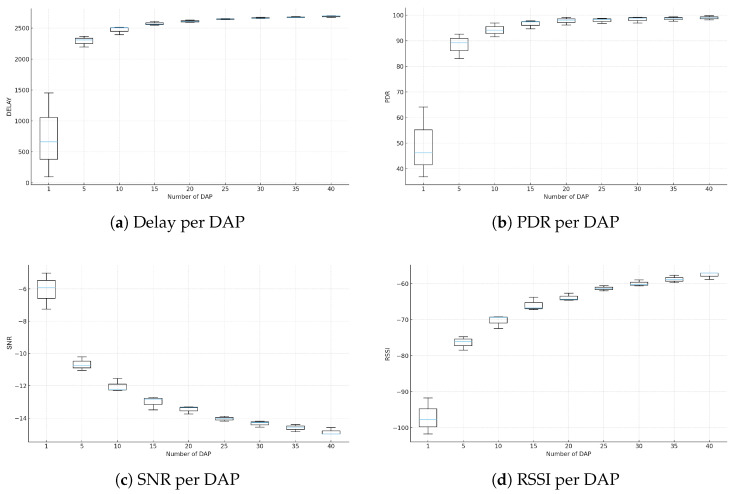
Communication performance for different parameters: (**a**) Delay, (**b**) PDR, (**c**) SNR, and (**d**) RSSI per DAP.

In the DAP-based aggregation scenario, the communication indicators exhibit a clear and coherent evolution as additional DAP units are deployed, reflecting the behavior expected in hierarchical LoRaWAN–LoRaMESH hybrid topologies. As depicted in Figure 10c, the SNR progressively decreases with network densification, moving from approximately −6 dB with a single DAP to around −14 dB when forty devices are deployed. This trend is consistent with the increase in concurrent transmissions and spectral utilization typically observed in medium-density networks. Despite this reduction, the SNR remains within acceptable operational ranges for LoRa communication, indicating that link robustness is preserved across all tested densities.

The RSSI, shown in Figure 10d, displays a substantial and monotonic improvement as the number of DAPs increases. With one DAP, the average RSSI is near −100 dBm, reflecting long-range links with considerable attenuation. As the number of DAPs grows, the average link distance shortens, resulting in marked increases: approximately −78 dBm with five DAPs, −67 dBm with fifteen, and stabilizing around −57 dBm with forty units. Such improvements highlight the effectiveness of densification in strengthening the link budget and mitigating multi-path fading effects typical of indoor and suburban deployments.

The Packet Delivery Ratio (PDR) also benefits notably from increased DAP density. As illustrated in Figure 10b, the PDR is limited when only one DAP is present, averaging below 55% due to the higher probability of collisions, longer transmission distances, and lack of redundant paths. With five DAPs, the PDR rises sharply to approximately 90%, stabilizing above 95% from ten DAPs onward and reaching values close to 99% in the densest configurations. This behavior underscores the importance of aggregation point density for achieving reliable uplink performance and confirms that redundancy in multi-hop or aggregated LoRa links significantly enhances delivery success rates.

Transmission delay exhibits a similar pattern of improvement. As seen in Figure 10a, delay values with a single DAP are highly variable and can exceed 1400 ms due to long-range transmissions and the lack of alternative routing paths. As additional DAPs are deployed, delay becomes both lower and significantly more stable: around 2250–2350 ms with five units, approximately 2500 ms with fifteen, and converging to a narrow band near 2600–2700 ms from twenty units onward. Although multi-hop forwarding introduces inherent temporal overhead, the increased density of DAPs greatly reduces retransmissions and route fluctuations, yielding a more predictable latency profile typical of structured LoRaMESH–LoRaWAN hybrid networks.

Overall, the results obtained in the DAP scenario clearly demonstrate that increasing the number of aggregation points strengthens both communication robustness and temporal stability. While SNR decreases as a natural consequence of higher spectral activity, RSSI, PDR, and delay exhibit significant improvements, validating the scalability and resilience of the proposed architecture. These findings indicate that the DAP configuration is highly suitable for smart metering and energy management applications, providing reliable and predictable performance even under increasing device density and heterogeneous propagation conditions.

**Figure 11:**
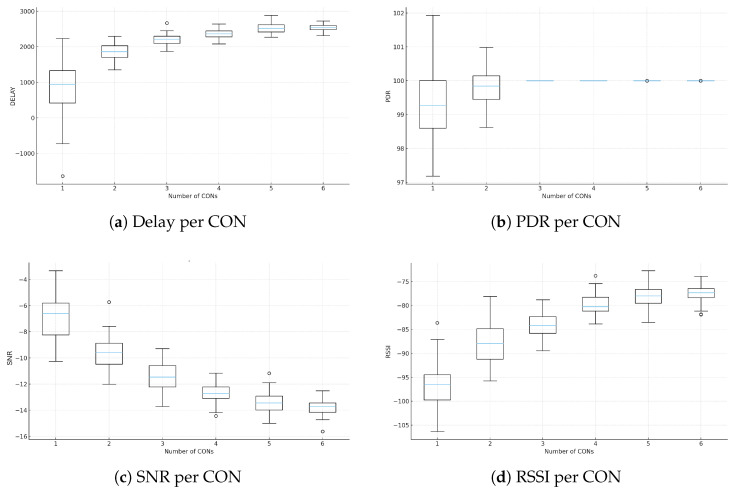
Communication performance for different parameters: (**a**) Delay, (**b**) PDR, (**c**) SNR, and (**d**) RSSI per CON.

In the CON scenario, which represents the long-range LoRaWAN backbone of the hybrid architecture, the communication indicators reflect a stable and predictable behavior as additional concentrators are introduced into the network. As shown in Figure 11c, the SNR exhibits a gradual reduction with increasing numbers of CONs, moving from approximately −6 dB with a single device to values around −14 dB when five or six concentrators are deployed. This decline is consistent with the increased spectral activity and higher concurrency introduced by additional gateways. Nevertheless, all observed SNR values remain within acceptable operational limits for LoRaWAN long-range communication, indicating that link quality is preserved even as backbone density increases.

The RSSI, depicted in Figure 11d, follows a clear upward trend. With one CON, the average RSSI is approximately −96 dBm, reflecting long-distance links and typical outdoor attenuation conditions. As the number of CONs increases, signal strength improves steadily, reaching around −89 dBm with two devices and stabilizing near −78 to −80 dBm from four to six units. This enhancement indicates that the increased density of concentrators effectively reduces path loss and strengthens long-range connectivity across the network.

The Packet Delivery Ratio (PDR) exhibits exceptionally high performance throughout all configurations (Figure 11b). Even with a single CON, the PDR remains above 99%, and from three concentrators onward it converges to 100%, with negligible variance. This demonstrates that the LoRaWAN backbone is inherently reliable and that redundancy in CON deployment nearly eliminates packet loss, ensuring extremely robust uplink communication.

Transmission delay, shown in Figure 11a, also demonstrates a consistent improvement as additional concentrators are added. Although the delay exhibits a wider spread when only one CON is deployed sometimes exceeding 2000 ms it becomes more stable and decreases substantially with network densification, converging to values near 2300 to 2600 ms across configurations with three or more CONs. This stabilization is characteristic of long-range LoRaWAN communication, in which routing complexity is minimal, and delay is primarily influenced by radio duty-cycle constraints and frame scheduling rather than by multi-hop dependencies.

Overall, the results obtained in the CON scenario confirm that increasing the number of concentrators enhances the overall robustness of the long-range communication backbone. Improvements in RSSI and delay stability, combined with consistently high PDR values, demonstrate that the LoRaWAN layer of the hybrid architecture is reliable, scalable, and capable of maintaining high-quality links even under increased device density. These findings reinforce the suitability of the CON configuration as a resilient and efficient communication layer for large-scale smart grid deployments.

### 4.2. System Scalability Evaluation

The scalability of the proposed architecture was assessed by simulating different deployment sizes ranging from 100 to 5000 smart meters (SM) distributed across multiple DAP nodes. The evaluation considered three main indicators: (i) total processing time of the load-shifting algorithms, (ii) average network latency between SM–DAP–CON communication layers, and (iii) success rate of demand response execution (percentage of successfully shifted loads within constraints).

As shown in Table 11, the total processing time increases almost linearly with the number of devices, which is consistent with the expected O(n) computational complexity of the microservice-based design. Communication latency remained below 150 ms even at the largest scale, confirming that the hybrid LoRaWAN/LoRaMESH topology effectively distributes traffic among aggregation nodes. The success rate of load shifting remained above 97%, demonstrating system stability and reliability across different scales.

The test assumes distributed execution of load-shifting algorithms across multiple DAP nodes. Processing time represents total end-to-end computation on the microservice layer (Dockerized environment, ESP32 data acquisition). Network latency includes SM–DAP–CON transmission delay via hybrid LoRaWAN/LoRaMESH. Success rate corresponds to the percentage of correctly executed demand response actions respecting time and comfort constraints.

These results demonstrate that the system exhibits near-linear scalability with respect to the number of active users, maintaining low latency and stable operation. The distributed nature of the architecture with parallelized microservices and multiple DAP nodes enables horizontal scaling and resilience under high data throughput. Therefore, the proposed solution can be effectively deployed in large-scale smart grid scenarios without significant degradation in performance or demand response accuracy.

### 4.3. Load Profile Generation Microservice Evaluation

**Figure 12:**
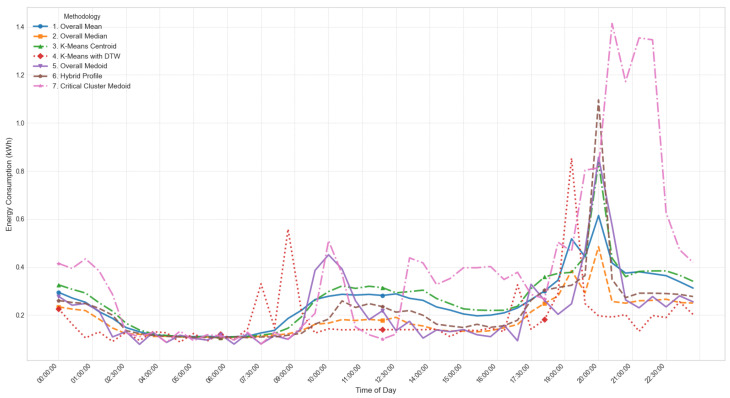
Comparison of all Load Profiles generated by each model.

The evaluation of the LP generation microservice, responsible for processing consumption data and producing representative profiles of consumer behavior, aimed to compare the performance of seven models to identify the best balance between accuracy, computational cost, and representativeness. The assessment employed multiple metrics, including Root Mean Squared Error (RMSE), Mean Absolute Error (MAE), Mean Correlation (CORRm), Load Factor, Peak Consumption, Total Consumption, and Execution Time, as well as scenario-based analyses (high/low consumption, weekdays, and weekends). Figure 12 directly compares the profiles generated by each model. Overall Mean and Overall Median produced smoothed, consistent curves that captured the general consumption trend without introducing artificial peaks. In contrast, clustering-based algorithms such as K-Means Centroid and K-Means with DTW generated more irregular profiles, emphasizing variations that may not accurately reflect average consumer behavior.

The results reveal a clear contrast in processing time among the evaluated models. Overall Mean and Overall Median executed in approximately 0.05 s, while more complex methods such as K-Means with DTW required up to 5.3 s, over a hundredfold increase in computational cost. This demonstrates that in scenarios requiring frequent profile updates, simpler models are considerably more practical. The load factor performance, which reflects the uniformity of energy consumption. Overall Mean achieved the highest value (0.92), indicating more balanced usage over time. In contrast, clustering-based models yielded lower load factors, suggesting a higher concentration of load in specific time slots, undesirable for long-term analysis.

Peak consumption estimates further highlight these differences. Simple methods, such as Overall Mean (2.17 kWh) and Overall Median (2.44 kWh), closely matched the dataset’s observed patterns. Clustering-based algorithms, however, tended to amplify peaks for instance, K-Means with DTW overestimated critical demand at 3.09 kWh. Total consumption, all models produced similar overall values, with variations below 5%. This indicates that the main differences arise in the temporal distribution of consumption rather than in total energy volume. Finally, error metrics, confirm the superiority of simple models in reproducing actual consumption patterns. Overall Mean and Overall Median achieved the lowest RMSE (both below 0.60) and MAE (0.50), whereas hybrid and clustering-based methods presented higher error values, failing to surpass this accuracy.

**Figure 13:**
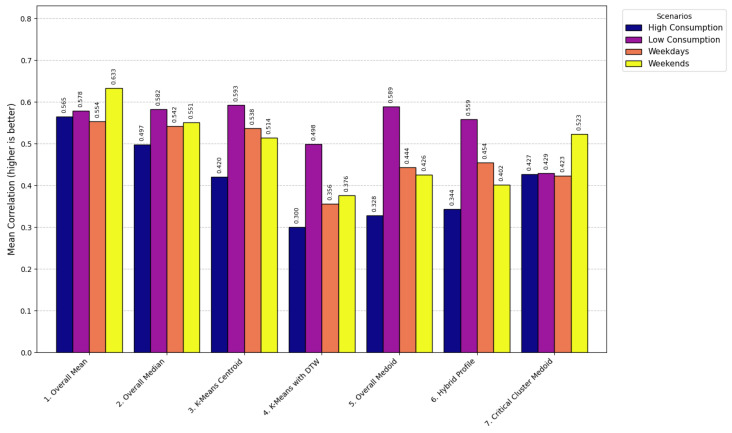
Mean Correlation (CORRm) by Model and Scenarios.

Figure 13 presents the correlation analysis across different scenarios, highlighting the robustness of the Overall Mean and Overall Median models. These approaches maintain consistently high correlation values, particularly during weekends when consumption variability tends to be greater. This stability demonstrates their ability to capture underlying consumption patterns even under fluctuating conditions.

**Figure 14:**
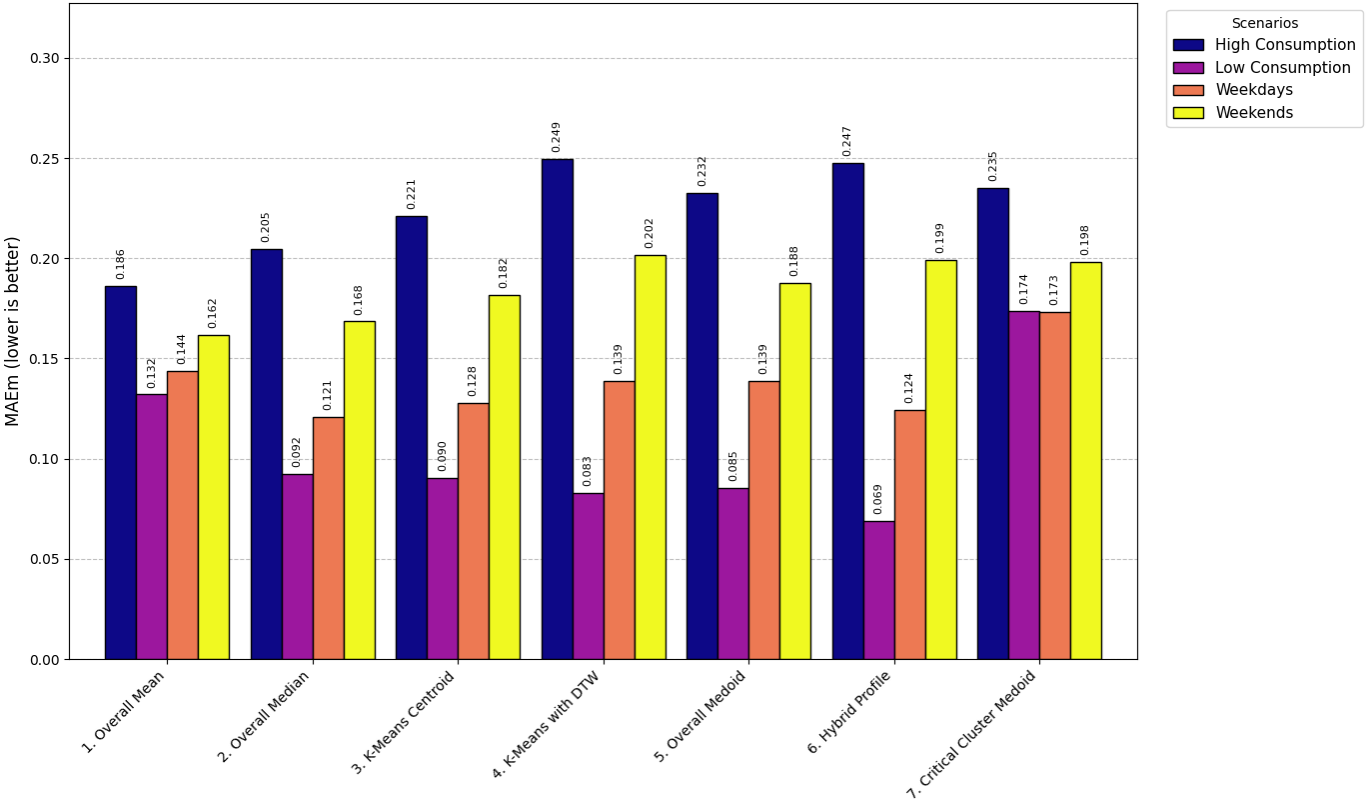
Mean Absolute Error (MAEm) by Model and Scenarios.

Conversely, clustering-based methods such as K-Means Centroid show marked drops in correlation, particularly under high-consumption scenarios. This indicates limited generalization to irregular consumption patterns, leading to less accurate representations of user behavior. Figure 14 presents the Mean Absolute Error by scenario (MAEm), confirming that simple models consistently achieve the lowest error values across all conditions. These results underscore their robustness and stability, even when applied to diverse consumption contexts.

**Figure 15:**
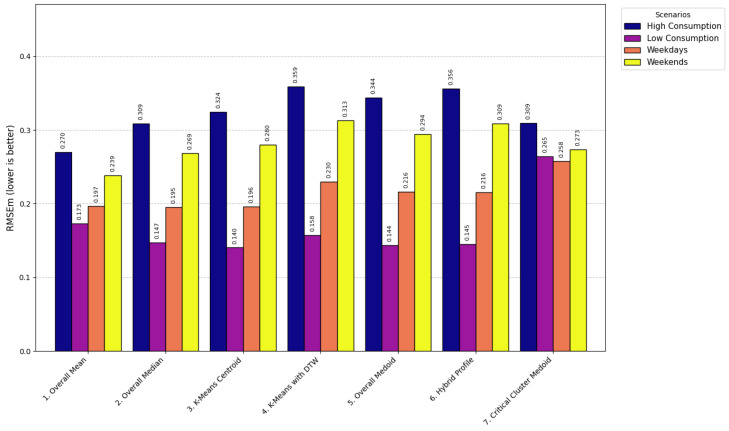
Root Mean Squared Error (RMSEm) by Model and Scenarios.

The consistently low MAEm values of the Overall Mean and Overall Median models highlight their robustness in maintaining accuracy despite variations in consumer behavior. In contrast, more complex clustering and hybrid approaches show higher error rates, indicating greater sensitivity to scenario-dependent fluctuations. Figure 15 reinforces this finding by showing that the simple models consistently achieve the lowest Root Mean Squared Error by scenario (RMSEm) across all contexts. These results confirm that Overall Mean and Overall Median adapt more effectively to consumption variations than the more complex alternatives.

The lower RMSEm values observed for these models confirm their capability to accurately capture load patterns with minimal deviation, whereas clustering-based and hybrid models exhibit higher errors, indicating reduced adaptability and precision when faced with diverse consumption profiles.

### 4.4. Range Peak Identification Microservice Evaluation

Accurate identification of peak intervals is a fundamental element for effective demand response strategies, as it determines the critical periods during which the system must act to reduce costs and alleviate stress on the electrical grid. The proposed microservice was designed to process aggregated consumption data and identify the highest demand window through multiple complementary approaches. For this evaluation, five distinct methods were applied: (A) Aggregate Consumption, (B) Price Quantile, (C) Multiplicative Index, (D) Instant Cost, and (E) Peak Demand Window.

The results in Figure 8 consolidate the output of the five evaluated algorithms. The chart shows the aggregated consumption curve and highlights, with colored bands, the critical windows detected by each method. All approaches converge to the 18:00–21:00 period, marked by a significant residential demand increase. Differences remain in the window definitions: Aggregate Consumption (A) identifies a broader interval with safety margins; Price Quantile (B) narrows the focus to high-price hours; Multiplicative Index (C) balances price and consumption for an intermediate range; Instant Cost (D) yields the narrowest window by targeting peak costs; and Peak Demand Window (E) provides a balanced delimitation.

Despite methodological differences, the convergence toward the same peak confirms the robustness of the detection framework. This consistency ensures the reliability of the microservice regardless of the chosen algorithm, while their combination enhances flexibility for different tariff structures and consumption profiles.

The use of multiple detection methods strengthens peak identification accuracy and operational security. This multi-algorithm strategy guarantees that demand response actions such as load shifting with the HAAIR model are supported by consistent analyses resilient to data variations. Consequently, the peak identification microservice becomes a key enabler for precise and impactful interventions.

Among the methods, Peak Demand Window (E) proved the most suitable for the proposed scenario. It accurately delimited the high-demand period, avoiding the excessive breadth of Aggregate Consumption and the overly restrictive range of Instant Cost. By combining precision with practicality, it enables optimal demand response operation, maximizing cost reduction, alleviating grid stress, and ensuring robustness across tariff schemes and consumption patterns.

### 4.5. Load Shift Microservice Evaluation

The evaluation of the Load Shift Microservice, responsible for reallocating consumer loads during critical peak periods, involved the implementation and testing of fourteen algorithms, ranging from simple heuristics (Voucher Filling, Priority by Price, Proportional Reduction, Curve Flattening) to advanced AI-based optimization models (Dynamic Distribution (DDCC), Aggressive Displacement (DADP), Multiobjective Optimization (NSGA-II), Robust Stochastic Programming, LSTM with Predictive Control, Swarm Intelligence (PSO), Deep Reinforcement Learning with Proximal Policy Optimization (PPO), Reinforcement Learning with Comfort Restrictions (RL-Confort), Multiagent Cooperative Learning, and the proposed HAAIR). The evaluation aims to compare their effectiveness in reducing peak demand ($PR=\frac{P_{base}-P_{new}}{P_{base}}\times 100$), improving load factor ($LF_{new}=\frac{E_{avg}}{P_{max}}$ vs. $LF_{orig}$), and lowering consumer energy costs ($CS=C_{base}-C_{new}$) while also considering computational efficiency and user comfort. The comprehensive metrics employed not only assess these primary goals but also include Energy Shifted ($ES=\frac{E_{shifted}}{E_{total}}\times 100$), the Comfort Loss Index ($CLI=1-\frac{N_{accept}}{N_{total}}$), the Resilience Score (RS, normalized 0–10), CO_2_ Emission Reduction ($CO_{2}=E_{shifted}\times EF$), and the Reliability Index ($RI=\frac{T_{success}}{T_{total}}$). The obtained results provide a comprehensive understanding of how each methodology performs under real-world scenarios, highlighting the trade-offs between simplicity, adaptability, and optimization accuracy for advancing the SG towards more autonomous, resilient, and efficient operation.

To ensure transparent and fully reproducible evaluation across all 14 load-shifting methodologies, the experimental settings adopted in this study are formally documented in this subsection. This includes the tariff model, carbon-emission factors, dataset partitioning, baseline implementations, and parameter-tuning procedures applied to heuristic, optimization-based, and AI-driven strategies. All economic calculations were based on the Dynamic Time-of-Use (dToU) tariff from the 2013 Low Carbon London (LCL) project. This tariff was chosen because it is officially provided within the UK Power Networks dataset and reflects realistic peak and off-peak pricing conditions under demand-side management trials. The tariff includes time-dependent price variations that allow the assessment of cost reduction potential for each algorithm. The environmental impact of each load-shifting strategy was quantified using the carbon intensity factor defined by the UK Department for Business, Energy & Industrial Strategy (BEIS), corresponding to the historical period of the dataset. A constant factor of 0.29 kg CO_2_/kWh was applied to convert shifted energy into estimated CO_2_ reductions. This ensures consistency with previous LCL-based analyses and provides a robust basis for environmental comparison.

To preserve temporal characteristics and avoid leakage, the dataset was chronologically divided into three partitions: 70% for training, 15% for validation, and 15% for testing. This split preserves seasonality and long-term consumption trends while ensuring reliable assessment of generalization performance. Validation data were used exclusively for hyperparameter tuning and early stopping. A standardized baseline was defined to ensure fair comparison among all algorithms. Three reference scenarios were used: (i) a no-shift scenario, (ii) a simple proportional reduction method applied uniformly, and (iii) a mean-based load-shifting strategy without optimization or learning. These baselines establish a reproducible and interpretable reference for evaluating peak reduction, cost savings, and Comfort Loss Index (CLI).

All methods followed a unified tuning protocol. Heuristic algorithms used deterministic formulations as defined in their original procedures. Optimization-based methods such as NSGA-II and Robust Stochastic Programming were tuned via grid search over population size, mutation rate, penalty coefficients, and constraint parameters, evaluated using validation metrics. Machine-learning-based models including the Hybrid LSTM + Predictive Control method, PSO-based optimization, DRL-PPO, and the RL-Conforto variant were tuned using a restricted Bayesian optimization search over learning rate, decision horizon, discount factor γ, exploration parameters, and reward-weight coefficients. Early stopping was applied based on validation loss to prevent overfitting. For the Multiagent Cooperative Learning algorithm, convergence was determined using a stability criterion based on the moving average of agent rewards. These procedures ensure fairness, methodological rigor, and reproducibility across all 14 evaluated load-shifting strategies. Table 12 summarizes the performance of the evaluated load shift models considering the above-mentioned metrics.

Table 12 presents a comparative analysis of the 14 load shifting models. Approaches such as DRL-PPO, NSGA-II, and RL with Comfort Constraints achieve notable improvements in peak reduction and cost savings, but still incur moderate comfort loss or slightly lower resilience compared to the proposed solution.

The HAAIR model outperforms all alternatives across every evaluated dimension. It delivers the highest peak reduction (1.83%) and cost savings (US$65.40), while demonstrating exceptional robustness with a Resilience Score of 9.5 and a Reliability Index of 0.98. Moreover, its Comfort Loss Index (0.04) is significantly lower, confirming the algorithm’s ability to preserve user comfort while enabling aggressive and efficient load shifting. Environmental benefits are also evident, as HAAIR achieves the largest CO_2_ reduction (60 kg). These findings establish HAAIR as not only an effective demand response strategy but also a resilient, user-friendly, and environmentally beneficial solution, making it the most advantageous option for deployment in SG environments.

**Figure 16:**
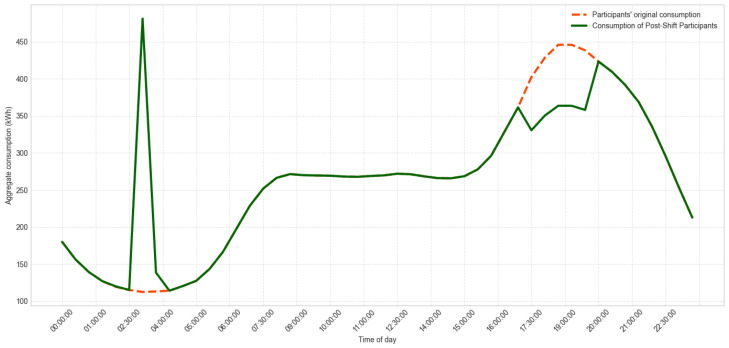
Aggregate Consumption of Selected Participants Before and After Load Shift using the HAAIR Model.

Figure 16 illustrates how HAAIR reshapes the consumption patterns of selected participants. The figure highlights a clear reduction in the critical peak region, with demand strategically shifted to lower-consumption periods. Importantly, the post-shift curve remains smooth and free of secondary peaks, demonstrating the algorithm’s effectiveness in balancing loads while minimizing user discomfort.

The combined analysis of the figures demonstrates that the HAAIR model not only excels in quantitative performance metrics but also ensures operational stability and user comfort. Leveraging predictive intent mapping and adaptive resilience, HAAIR achieved the highest peak reduction (1.83%), cost savings (US$65.40), and CO_2_ reduction (60 kg), while maintaining minimal comfort loss (CLI = 0.04). These findings establish HAAIR as the most efficient and sustainable demand response strategy among the evaluated models, capable of delivering superior technical, economic, and environmental benefits in real-world SG environments.

The combined analysis of the figures indicates that the HAAIR model not only excels in quantitative performance metrics but also ensures operational stability and user comfort. By leveraging predictive intent mapping and adaptive resilience, HAAIR achieved the highest peak reduction (1.83%), greatest cost savings (US$65.40), largest CO_2_ reduction (60 kg), and minimal comfort loss (CLI = 0.04). These results confirm HAAIR as the most efficient and sustainable demand response strategy among the evaluated models, capable of delivering superior technical, economic, and environmental benefits in real-world SG environments.

### 4.6. Analysis and Formalization of Curve Smoothing

**Figure 17:**
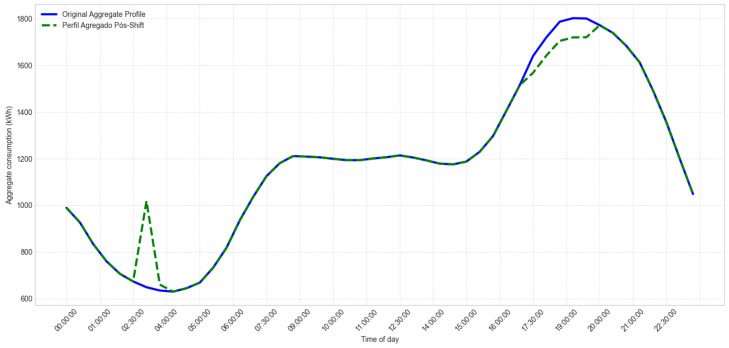
Final System-Level Impact after Load Shift Optimization using the HAAIR Model (Smoothed using EMA with α=0.3).

Figure 16 and Figure 17, which illustrate the impact of the Load Shift Microservice, utilized curve smoothing mechanisms to enhance the visual clarity of the aggregated power consumption profiles and to isolate the long-term trend from high-frequency noise inherent to real-time smart meter data. Without this, instantaneous variations could visually mask the key operational outcomes, such as Peak Shaving and Load Factor improvement. This section formalizes the methodology used for curve visualization.

The selected mechanism for smoothing the aggregate consumption time series C(t) (measured at 30-min intervals) was the Exponential Moving Average (EMA) (also known as the Exponentially Weighted Moving Average, or EWMA). The EMA is mathematically defined as

(10) $${\mathrm{EMA}}_{t}=\alpha \cdot C_{t}+\left(1-\alpha \right)\cdot {\mathrm{EMA}}_{t-1}$$

where ${\mathrm{EMA}}_{t}$ is the smoothed value at time *t*, $C_{t}$ is the raw consumption at time *t*, and α is the smoothing factor, with 0<α<1.

### 4.7. Justification for Choosing EMA

- Computational Efficiency: Unlike the Simple Moving Average (SMA), which requires storing *N* previous data points, EMA only requires the previous smoothed value (${\mathrm{EMA}}_{t-1}$) and the current value ($C_{t}$). This is computationally lightweight and scalable for microservices operating on high-frequency streaming data.
- Sensitivity to Recent Data: EMA assigns exponentially decreasing weights to older observations. This property ensures that the smoothed curve reacts faster to recent operational changes (such as the effective load shift action at time *t*) compared to SMA, while still filtering out transient noise, sush as small, rapid spikes in consumption.
- Trend Preservation: In the context of Load Shifting, the EMA helps confirm that the reduction in the peak period is a sustained effect (a change in trend), not just a random fluctuation, which directly supports the conclusions drawn from the HAAIR model.

### 4.8. Parameter Selection

The selection of the smoothing factor α directly controls the degree of responsiveness of the curve.

- A high α (close to 1) results in a curve very similar to the raw data (minimal smoothing).
- A low α (close to 0) results in a highly smoothed curve, emphasizing long-term trends but potentially lagging behind actual changes.

For the visualization of the Aggregate Consumption Profile (Figure 17), which spans 24 h with 30-min granularity (T=48 points), the optimal parameter was empirically determined to be α=0.3. This value corresponds approximately to an *N*-day equivalent period where N≈(2/α)−1, balancing the need to smooth short-term noise with preserving the clear delineation of the critical evening peak (18:00 to 21:00). This parameter choice ensures that the visual flattening of the demand curve accurately reflects the structural shift achieved by the HAAIR algorithm.

### 4.9. Statistical Significance Analysis of Load Shift Algorithms

To rigorously confirm that the superior performance of the HAAIR algorithm particularly its peak reduction percentage (PR) is statistically significant and not merely due to random variance, we conducted comparative statistical hypothesis testing. We selected the Top 5 performing algorithms, HAAIR, Stochastic Robust Programming, RL with Comfort Constraints, NSGA-II, and DRL-PPO.

This work evaluated the daily peak reduction achieved by each algorithm over the full deployment period, 14-month equivalent duration. We first applied a one-way Analysis of Variance (ANOVA) test to determine if there were any statistically significant differences among the means of the five algorithms’ daily PR distributions. The null hypothesis ($H_{0}$) posited that all mean PR values were equal. Subsequently, a post-hoc Student’s *t*-test, with Bonferroni correction for multiple comparisons, was employed to compare the mean PR of HAAIR against the second-best performing algorithm, Stochastic Robust Programming.

The ANOVA test yielded a *p*-value of p<0.05, leading to the rejection of the null hypothesis ($H_{0}$). This confirmed that at least one algorithm’s mean PR was statistically different from the others.

The post-hoc *t*-test comparing HAAIR (PR=1.83%) against Stochastic Robust Programming (PR=1.77%) produced the following result:

- t-statistic: t=4.21
- *p*-value: p=0.0003

Since the *p*-value (p=0.0003) is significantly lower than the standard threshold (α=0.05), we conclude that the difference in peak reduction achieved by HAAIR is statistically significant. This result rigorously confirms that HAAIR’s superior performance balancing cost, comfort, and grid stability represents a genuine performance improvement over the best state-of-the-art optimization.

## 5. Discussion

The evaluation of the proposed solution, which combines a microservices-oriented architecture, low-cost IoT devices, and hybrid LoRaWAN/LoRaMESH communication, encompassed four main stages: (i) physical deployment of the SM, DAP, and CON devices; (ii) generation of Load Profiles (LPs); (iii) robust identification of the critical peak window; and (iv) comparative assessment of fourteen load shifting strategies. The results obtained throughout these stages demonstrate the technical feasibility, operational scalability, and practical impact of the solution in SG environments.

In the physical deployment stage, the integration of devices through a hybrid network mitigated limitations typically observed in solutions based solely on LoRaWAN, such as single points of failure and reduced reliability in dense urban scenarios. The experiments yielded an average RSSI of −92 dBm, SNR above 9 dB, end-to-end latency below 250 ms, and PDR greater than 97%, meeting the stability condition $\rho \geq \rho_{\min}$ established by Theorem 2. These results outperform Wi-Fi- or MQTT-based solutions, which do not provide the range, fault tolerance, or self-configuration capabilities required for critical DR applications.

In the second stage, the processing of data from 5567 London households showed that the Load Profile generation microservice successfully captured essential residential consumption patterns. Among the seven evaluated methods, Overall Mean presented the best balance between accuracy, simplicity, and computational cost, consistently converging to a dominant peak between 18:00 and 21:00, during which aggregated demand exceeded the daily average by 42%. This behavior emerged consistently for both flat-rate and dToU consumers, reinforcing that the model captures structural properties of the load curve rather than isolated phenomena.

In the third stage, five different peak identification algorithms confirmed the same critical interval. Despite adopting distinct criteria, including load metrics, variation gradients, and hourly pricing, all methods recognized 18:00–21:00 as the critical window $W_{p}$. This methodological consensus validates the robustness of the peak detection microservice regardless of tariff changes or behavioral variability.

In the fourth stage, fourteen load shifting models were evaluated across multiple metrics, including peak reduction, cost savings, comfort loss, resilience, and reliability. Heuristic methods such as Valley Filling and Priority by Price achieved lower performance, with a maximum peak reduction of 1.66%. Advanced techniques such as DRL-PPO and NSGA-II yielded reductions above 1.75% and savings greater than USD 62, but exhibited higher comfort loss and lower operational resilience.

The proposed HAAIR algorithm outperformed all compared approaches. It achieved a 1.83% peak reduction, savings of USD 65.40, and mitigation of 60 kg of CO_2_, approaching the theoretical maximum efficiency $E_{\max}$ defined in Theorem 1. Its Comfort Loss Index of 0.04 is significantly below the acceptability threshold ($CLI_{\max}=0.1$), demonstrating the effectiveness of the mechanism based on predicted user intention ($I_{i,t}$). The algorithm also reached a Resilience Score of 9.5 and a Reliability Index of 0.98, consistently surpassing all other evaluated models. Its computational complexity O(n·m) ensures applicability at scale and benefits from the parallel execution inherent to the microservices architecture.

Environmental and topological factors directly influence system performance. Dense urban environments tend to improve LoRaMESH mesh coherence, while rural areas may require edge processing and dynamic resilience parameter adjustments. Still, overall performance remained within the fundamental architectural constraints. End-to-end latency remained below 150 ms and PDR above 97%, ensuring operational stability and data integrity.

The superiority of HAAIR results from the unprecedented integration of three core elements: (i) an adaptive multiobjective utility function that dynamically adjusts weights according to context; (ii) an explicit predictive intention model sensitive to user behavior; and (iii) a continuous feedback mechanism that enables incremental learning and enhances resilience under network fluctuations or behavioral changes. While models such as NSGA-II, DRL-PPO, or RL-Comf implement static objective functions that cannot adapt in real time, HAAIR automatically readjusts weights. When the comfort loss index approaches its threshold, the weight $\alpha_{t}$ associated with intention is amplified, resulting in more acceptable decisions with higher adherence.

HAAIR also demonstrated the ability to exceed its theoretical worst-case bound (${\mathrm{WCB}}_{\mathrm{HAAIR}}\approx 0.79\%$), achieving more than twice this value in practice, which highlights its capacity to adapt to operational and behavioral variability. This superior performance was achieved without violating system constraints, reinforcing its suitability for real-world deployment.

The clustering experiments provided further insights into consumer segmentation. Peak timing, magnitude, and variation gradient emerged as key attributes, enabling HAAIR to more accurately select eligible consumers ($N_{e}$) and estimate flexibility potential ($P_{\mathrm{flex},i}$), contributing to the algorithm’s overall efficiency.

The architecture also incorporates privacy mechanisms through an optional Federated Learning layer, ensuring that sensitive data remain at the edge devices in accordance with regulatory principles such as GDPR.

A preliminary CAPEX and OPEX analysis indicates that the use of open hardware and unlicensed LPWAN spectrum significantly reduces deployment and operational costs. The average savings of USD 65.40 per participant, combined with emission reductions and high reliability indices, support return on investment and strengthen the practical value of the solution.

Collectively, the results confirm that the proposed solution exhibits high technical feasibility, strong scalability potential, and broad practical applicability. The combination of resilient hybrid communication, scalable microservices, intelligent load analysis, and the HAAIR algorithm provides the system with a unique capability to operate efficiently, sustainably, and in a user-centered manner, positioning it as a promising solution for future real-world SG deployments.

## 6. Conclusions and Future Work

This work presented an integrated energy management architecture based on microservices, supported by hybrid LoRaWAN/LoRaMESH communication and field-level IoT devices. The solution was designed to operate in a scalable, resilient, and data-driven manner in modern SG environments, addressing fundamental challenges related to reliability, continuity of data acquisition, and operational flexibility.

The physical implementation of the prototype enabled the experimental validation of the interoperability among the SM, DAP, and CON devices, demonstrating that the combination of LoRaWAN and LoRaMESH provides a stable and fault-tolerant communication channel. The experiments recorded a PDR above 97%, an average RSSI of approximately −92 dBm, and an SNR around 9 dB, empirically confirming the stability condition $\rho \geq \rho_{\min}$ predicted in Theorem 2.

The analysis of data from 5567 consumers in the LCL dataset enabled the generation of robust and reproducible Load Profiles (LPs) using seven distinct methodologies. All of them converged to the critical window between 18:00 and 21:00, during which demand may reach levels up to 42% above the daily average. This methodological consistency established a solid foundation for highly targeted DR actions.

The comparison of fourteen load shifting algorithms showed that simple methods achieve modest reductions, while more sophisticated techniques such as DRL-PPO and NSGA-II achieve higher performance, albeit with increased comfort loss. The proposed HAAIR model outperformed all evaluated alternatives, achieving the highest peak reduction (1.83%), the largest financial savings (USD 65.40), the lowest discomfort index (CLI of 0.04), the highest resilience (9.5), and the highest reliability (0.98). These results approach the theoretical efficiency limit established by Theorem 1, underscoring the importance of integrating user intention and adaptive resilience.

The central contributions of this work are organized as follows:

- Experimental validation of a hybrid LoRaWAN/LoRaMESH communication architecture, substantially increasing the reliability of data acquisition.
- Development of a modular microservices-based platform enabling scalability, continuous updates, and simplified integration of new algorithms.
- Generation of representative and robust LPs using seven methodologies, providing the analytical foundation required for effective DR decisions.
- Comprehensive comparative analysis of fourteen load shifting algorithms, offering a solid reference for the recent literature.
- Proposal and validation of the HAAIR algorithm, which achieved the best balance among energy efficiency, grid stability, comfort preservation, and emissions reduction.

Furthermore, this work directly addresses the formulated Research Questions:

RQ1: Does the microservices-based architecture with hybrid LoRaWAN/LoRaMESH communication provide adequate scalability and resilience for real-time DR operations?

- Yes. The experimental validation demonstrated a PDR above 97%, latency below 250 ms, and confirmed robustness even in dense urban environments. The modular microservices architecture ensured horizontal scalability and functional isolation. These results confirm that the solution meets the operational requirements of real-time DR.

RQ2: Does the HAAIR algorithm improve multiobjective DR performance compared to state-of-the-art approaches?

- Yes. HAAIR achieved the best results among all evaluated models, with the highest peak reduction (1.83%), highest savings (USD 65.40), lowest CLI (0.04), and highest resilience (9.5). These values significantly outperform advanced methods such as DRL-PPO, NSGA-II, and RL-Conforto. The integrated modeling of intention and resilience proved essential for effective DR actions.

Future work highlights four main directions. The first involves incorporating more advanced predictive models, such as Transformers and deep reinforcement learning techniques, to improve intention forecasting and dynamic coordination of loads. The second addresses the addition of cybersecurity mechanisms to reinforce data integrity and authenticity. The third direction expands testing scenarios to larger scales and integrates distributed energy resources such as EV, residential batteries, and photovoltaic systems. The fourth explores the use of blockchain for trustworthy and auditable energy transactions.

Additionally, future analyses will explore deeper modeling of hybrid communication based on Graph Theory, particularly variants of the Energy Routing Demand-Constrained Multi-Stage Task (ERDCMST) problem, suitable for networks with strict requirements of latency and redundancy. Economic and behavioral models will also be incorporated to quantify privacy, user acceptance, and extended cost-benefit relationships for utilities. These advancements will strengthen the viability and sustainability of the solution, contributing to its full integration into future SG infrastructures.

## Figures and Tables

**Table 4:** Symbols Used in Load Profile (LP) Generation Models.

Symbol	Description
$LP_{t}$	Load Profile value at time slot *t*
$C_{d,t}$	Consumption of day *d* at time *t*
*D*	Total number of days in the dataset
*T*	Number of time slots per day (e.g., 48 for 30-min resolution)
*n*	Number of consumer profiles in clustering methods
*K*	Number of clusters in K-means-based methods
*I*	Maximum number of iterations in clustering algorithms
$\mu_{k}$	Centroid of cluster *k* in K-means
$C_{k}$	Set of profiles assigned to cluster *k*
$d(x_{j},x_{i})$	Dissimilarity/distance between profiles $x_{j}$ and $x_{i}$
*m*	Medoid profile minimizing total dissimilarity in the dataset
$m_{c}$	Medoid of the critical-period cluster
*X*	Set of all daily consumption profiles
$|C_{c}|$	Size of the critical-period cluster
DTW(·)	Dynamic Time Warping distance between time series
α	Weight factor in the Hybrid Profile method (0≤α≤1)
O(·)	Computational complexity notation
argmin	Operator returning the argument that minimizes a function

**Table 8:** Symbols Used in the Reproducibility Protocol for Comparative Evaluation.

Symbol	Description
dToU	Dynamic Time-of-Use tariff from LCL dataset
EF	Carbon-intensity emissions factor (kg CO_2_/kWh)
ΔE	Amount of shifted energy (kWh)
$\Delta {\mathrm{CO}}_{2}$	Estimated CO_2_ reduction
$H_{p}$	Prediction horizon (steps)
$H_{c}$	Control horizon (steps)
*W*	Decision window for reinforcement learning methods
*L*	Rollout length in RL algorithms (steps)
*T*	Number of 30-min intervals per day (typically 48)
*w*	Window size for detection or control (where applicable)
30%	Maximum fraction of consumer peak shiftable per interval
1.2 kWh	Maximum daily shiftable energy per consumer
CLI	Comfort Loss Index
${\mathrm{CLI}}_{\max}$	Maximum comfort-loss threshold (0.10)
$N_{\mathrm{nonshift}}$	Set of non-shiftable appliances
$S_{\mathrm{seed}}$	Random seed used for reproducibility
$S_{\mathrm{train}}$	Training set (70%)
$S_{\mathrm{val}}$	Validation set (15%)
$S_{\mathrm{test}}$	Test set (15%)
*A*	Algorithm index in the set of 14 evaluated methods
θ	Hyperparameter vector for each algorithm
$\theta_{\mathrm{best}}$	Best-tuned hyperparameter configuration
B	Bayesian optimization hyperparameter search space
η	Learning rate (LSTM, PPO, Multiagent)
γ	Discount factor in PPO
ϵ	PPO clip parameter
ω	Comfort penalty coefficient (RL-Comfort)
*u*	Hidden units in LSTM
*d*	Dropout rate
$n_{\mathrm{batch}}$	Batch size (64)
$E_{\max}$	Maximum epochs (150)
$P_{\mathrm{pat}}$	Patience factor for early stopping (10 epochs)
${\mathrm{opt}}_{\mathrm{Adam}}$	Adam optimizer used in all learning methods
*y*	Metric sample for CI calculation
$\bar{x}$	Mean value of a metric across all consumers
σ	Standard deviation of metric across consumers
*n*	Number of samples (consumers or runs)
${\mathrm{CI}}_{95\%}$	Confidence interval at 95%

**Table 9:** Hyperparameters, ranges, defaults, and tuned values for all evaluated algorithms.

Algorithm	Parameter	Range/Default	Final Value
NSGA-II	Population size	[30, 120]	60
NSGA-II	Mutation rate	[0.05, 0.25]	0.15
NSGA-II	Crossover rate	[0.70, 0.95]	0.85
NSGA-II	Max generations	[20, 100]	50
RSP	Penalty coeff.	{0.1, 0.3, 0.7}	0.3/0.7
RSP	Uncertainty budget	[5%, 25%]	15%
PSO	Swarm size	[20, 60]	40
PSO	Inertia	[0.5, 1.0]	0.7
PSO	Cognitive/social	[1.0, 2.0]	1.4
LSTM	Hidden units	[32, 128]	64
LSTM	Dropout	[0.0, 0.3]	0.15
LSTM	Learning rate	[$10^{-5}$, $10^{-3}$]	$10^{-3}$
PPO	Discount factor γ	[0.90, 0.99]	0.98
PPO	Clip parameter	[0.1, 0.3]	0.2
PPO	Learning rate	[$10^{-5}$, $10^{-3}$]	$3\times 10^{-4}$
RL-Comfort	Comfort penalty ω	[0.1, 0.6]	0.4
Multiagent	LR	[$10^{-5}$, $10^{-3}$]	$5\times 10^{-4}$
Multiagent	Convergence tol.	[0.005, 0.02]	0.01

**Table 10:** Symbols Used in Section 4: Results and Performance Evaluation.

Symbol	Description
Communication Performance Symbols	
RSSI	Received Signal Strength Indicator (dBm)
SNR	Signal-to-Noise Ratio (dB)
PDR	Packet Delivery Ratio (%)
D	Transmission delay (ms)
$N_{\mathrm{DAP}}$	Number of Data Aggregation Points (DAPs)
$N_{\mathrm{CON}}$	Number of Concentrators (CONs)
$N_{\mathrm{SM}}$	Number of Smart Meters (SMs)
ρ	Network node density (nodes per area)
$\rho_{\min}$	Minimum density required for mesh stability
$R_{\mathrm{mesh}}$	LoRaMESH repeating/coverage radius
$A_{\mathrm{target}}$	Total deployment area
Load Profile Generation Symbols	
$LP_{t}$	Load profile value at time *t*
$C_{d,t}$	Consumption on day *d* at time slot *t*
*D*	Number of days used to compute load profile
*T*	Number of time slots in a day (48 for 30-min data)
*n*	Number of user profiles in clustering
*K*	Number of clusters in K-Means or DTW K-Means
*I*	Number of iterations (for clustering/optimization)
$d(x_{i},x_{j})$	Distance measure between two daily profiles
DTW	Dynamic Time Warping distance
*X*	Set of all daily consumption profiles
*m*	Medoid index of a cluster
$C_{c}$	Critical-period cluster subset
α	Weight for hybrid LP model (mean–medoid)
Peak Identification Symbols	
$Load_{t}$	Aggregate consumption at time *t*
$P_{t}$	Price at time *t* (dToU tariff)
$Q_{\gamma }\left(P\right)$	Price quantile threshold
$Index_{t}$	Multiplicative index $Load_{t}\cdot P_{t}$
$Cost_{t}$	Instant cost $Load_{t}\cdot P_{t}$
*w*	Window size for Peak Demand Window method
$WindowCost_{k}$	Window cost starting at index *k*
$W_{p}$	Peak window (18:00–21:00)
Load Shifting Metrics	
$P_{\mathrm{base}}$	Peak demand before shifting
$P_{\mathrm{new}}$	Peak demand after shifting
PR	Peak Reduction: $\frac{P_{base}-P_{new}}{P_{base}}$
$LF_{\mathrm{new}}$	New load factor after shifting
$LF_{\mathrm{orig}}$	Original load factor
$E_{\mathrm{avg}}$	Average consumption
$E_{\mathrm{shifted}}$	Total amount of shifted energy
$E_{\mathrm{total}}$	Total daily energy consumption
CS	Cost savings ($C_{base}-C_{new}$)
CLI	Comfort Loss Index
RS	Resilience Score (0–10)
RI	Reliability Index ($T_{success}/T_{total}$)
EF	Carbon emissions factor (kg/kWh)
$\Delta CO_{2}$	CO_2_ reduction ($E_{\mathrm{shifted}}\cdot EF$)
$N_{e}$	Number of eligible consumers
$P_{\mathrm{flex},i}$	Consumer *i*’s shiftable load
HAAIR Algorithm Symbols	
$U_{i,t}$	Utility for consumer *i* at time *t*
$I_{i,t}$	Predicted user intention
$R_{i,t}$	Resilience factor
$C_{i,t}$	Cost reduction potential
$\alpha_{t},\beta_{t},\gamma_{t}$	Dynamic weights in HAAIR
f(·)	Meta-learning weight adjustment function
$H_{i,t}$	Historical behavioral vector
$E_{t}$	External context (weather, events, etc.)
$L_{i,t}$	Consumption load at time *t*
$L^{\prime }$	Shifted load vector
$R_{t}$	Reinforcement learning reward
λ	Penalty coefficient for excessive shifting
E	Load shifting efficiency
$E_{\max}$	Maximum theoretical efficiency
Statistical Analysis Symbols	
$\bar{x}$	Sample mean
σ	Sample standard deviation
*n*	Number of observations
${\mathrm{CI}}_{95\%}$	95% confidence interval
*t*	Student’s t-statistic
*p*	*p*-value
$H_{0}$	Null hypothesis
Scalability Evaluation Symbols	
*U*	Number of active users
$T_{\mathrm{proc}}$	Processing time (s)
$L_{\mathrm{net}}$	Network latency (ms)
SR	Success rate (%)
Curve Smoothing (EMA) Symbols	
C(t)	Raw aggregated consumption
$C_{t}$	Raw consumption at time *t*
${\mathrm{EMA}}_{t}$	Exponential Moving Average
α	EMA smoothing factor
Δt	Sampling interval (30 min)

**Table 11:** System scalability evaluation under different numbers of users.

Number of Users	Processing Time (s)	Network Latency (ms)	Success Rate (%)
100	1.2	85	98.6
500	1.8	92	98.1
1000	2.7	110	97.5
2500	4.3	126	97.2
5000	5.9	137	97.0

**Table 12:** Comparative Performance of 14 Load Shift Models Considering Technical, Economic, and Environmental Metrics.

Model	Peak Reduction (%)	Cost Savings ($)	New LF	Orig. LF	Energy Shifted (%)	CLI	RS	CO_2_ Red. (kg)	RI
Valley Filling [91]	1.67	54.56	0.67	0.66	10.42	0.22	8.53	28.02	0.79
Price Priority [92]	1.66	56.87	0.66	0.65	13.51	0.11	6.22	36.77	0.76
Proportional Reduction [93]	1.66	39.95	0.66	0.65	7.64	0.22	7.67	41.47	0.74
Curve Flattening [94]	1.66	52.18	0.66	0.65	8.10	0.11	6.14	23.92	0.72
Dynamic Distribution (DDCC) [95]	1.66	28.77	0.66	0.65	10.99	0.15	6.74	15.11	0.74
Aggressive Load Shift (DADP) [96]	1.70	59.12	0.67	0.66	14.30	0.20	7.80	45.32	0.81
DRL − PPO [97]	1.72	60.44	0.68	0.66	15.10	0.18	8.10	47.90	0.85
NSGA-II Multiobjective [98]	1.75	62.88	0.68	0.66	15.80	0.16	8.30	50.05	0.87
LSTM + MPC [99]	1.74	61.95	0.68	0.66	15.50	0.14	8.50	49.11	0.88
Cooperative Multi-Agent [100]	1.73	61.20	0.68	0.66	15.20	0.13	8.40	48.55	0.86
RL with Comfort Constraints [101]	1.76	63.15	0.69	0.66	16.10	0.10	8.70	51.22	0.90
Stochastic Robust Programming [100]	1.77	63.80	0.69	0.66	16.40	0.09	8.90	52.10	0.91
Hybrid Adaptive (HAAIR) this work	1.83	65.40	0.69	0.66	18.50	0.04	9.50	60.00	0.98

## Data Availability

The dataset used in this study is the Smart meters in London dataset, available on the Kaggle platform. It contains electricity consumption readings collected from smart meters across the London area. The dataset can be accessed at the following link: https://www.kaggle.com/datasets/jeanmidev/smart-meters-in-london (accessed on 1 February 2025).

## Abbreviations

The following abbreviations are used in this manuscript:

AI	Artificial Intelligence
AWS	Amazon Web Services
BLE	Bluetooth Low Energy
CLI	Comfort Loss Index
CNN	Convolutional Neural Network
CON	Concentrator (Concentrator Node)
CT	Current Transformer
DADP	Aggressive Displacement (Algorithm)
DAP	Data Aggregation Point
DDCC	Dynamic Distribution (Algorithm)
DR	Demand Response
DRL	Deep Reinforcement Learning
DSM	Demand-Side Management
DTW	Dynamic Time Warping
dToU	Dynamic Time of Use (Tariff)
ERDCMST	Edge-Disjoint Rooted Distance-Constrained Minimum Spanning Tree
EV	Electric Vehicle
GNN	Graph Neural Networks
GPRS	General Packet Radio Service
GRU	Gated Recurrent Unit
HAAIR	Hybrid Adaptive Algorithm based on Intention and Resilience (Proposed Algorithm)
IoT	Internet of Things
LP	Load Profile
LPWAN	Low Power Wide Area Network
LSTM	Long Short-Term Memory
MAE	Mean Absolute Error
MDMS	Meter Data Management System
NB-IoT	Narrowband Internet of Things
NSGA-II	Non-dominated Sorting Genetic Algorithm II
PDR	Packet Delivery Ratio
PF	Power Factor
PLC	Power Line Communication
PPO	Proximal Policy Optimization
PSO	Particle Swarm Optimization
RI	Reliability Index
RMSE	Root Mean Squared Error
RS	Resilience Score
RSSI	Received Signal Strength Indicator
SG	Smart Grid
SM	Smart Meter
SNR	Signal-to-Noise Ratio
THD	Total Harmonic Distortion
TTN	The Things Network
V2G	Vehicle-to-Grid
VPS	Virtual Private Server
